# Utilisation of mango seed husk for the production of phenolic compounds and glucose with C1184 enzyme preparation reveals the role of glucuronoyl esters in lignin–carbohydrate linkages in biomass recalcitrance

**DOI:** 10.1186/s40643-025-00989-z

**Published:** 2025-12-27

**Authors:** Mpho Stephen Mafa, Mamosela Marriam Mohotloane, Orbett Alexander, Mathapelo Hope Masilo, Anikó Várnai

**Affiliations:** 1 Carbohydrates and Enzymology Laboratory (CHEM-LAB), Department of Plant Sciences, University of the Free State, P.O. Box 339, Bloemfontein, 9300 South Africa; 2 Department of Chemistry, University of the Western Cape, Bellville, Cape Town, 7535 South Africa; 3 Faculty of Chemistry, Biotechnology and Food Science, Norwegian University of Life Sciences (NMBU), P.O. Box 5003, 1432 Aas, Norway

**Keywords:** CAZymes, Cellulose, Xylanase, Mannanase, Esterase, Mango seed husk, Lignin–carbohydrate complex

## Abstract

This study assessed mango seed husk (MSH) fractions for producing glucose and phenolic compounds using commercial enzymes. We focused on cleaving lignin–carbohydrate linkages, specifically feruloyl and glucuronoyl esters, to decrease biomass recalcitrance and enhance product extraction. For saccharification studies, we used Sigma’s C1184 cellulase from *Aspergillus niger*. Characterisation results of ground MSH using phloroglucinol and scanning electron microscopy revealed that it could be separated into a fine fraction, containing less lignin and cellulose fibres with parallel orientation, and a coarse fraction, with higher lignin content and cellulose fibres at an angled orientation. Activity assays and zymogram analysis of the C1184 preparation prior to saccharification studies revealed diverse CAZyme activities associated with distinct proteins, with xylanolytic activity dominating. Saccharification studies with ground MSH found that the C1184 preparation supplemented with feruloyl or glucuronoyl esterases was suitable for extracting phenolic compounds (0.4–1.7% w/w) from MSH while converting up to 20% of the total biomass as glucose. Interestingly, when replacing 50% (w/w) of the C1184 preparation with glucuronoyl esterase, glucose release nearly doubled from both MSH fractions. Additionally, phenolics attached to carbohydrates may be less condensed in the fine fraction, as all three esterases released three-to-five times more phenolics from the fine fraction compared to the coarse fraction with higher lignin content. Saccharification trials with alkali-pretreated ground MSH showed that the C1184 preparation supplemented with β-glucosidase produced low glucose levels (170–250 mg/g dry biomass) from the substrate after 24 h, even at 50 mg/g biomass protein loading. Overall, this work advances our understanding of the importance of lignin–carbohydrate linkages formed via glucuronoyl esters in biomass recalcitrance. Furthermore, our study corroborates the potential of MSH as a valuable feedstock for producing value-added products in the biorefinery sector.

**Graphical abstract:**

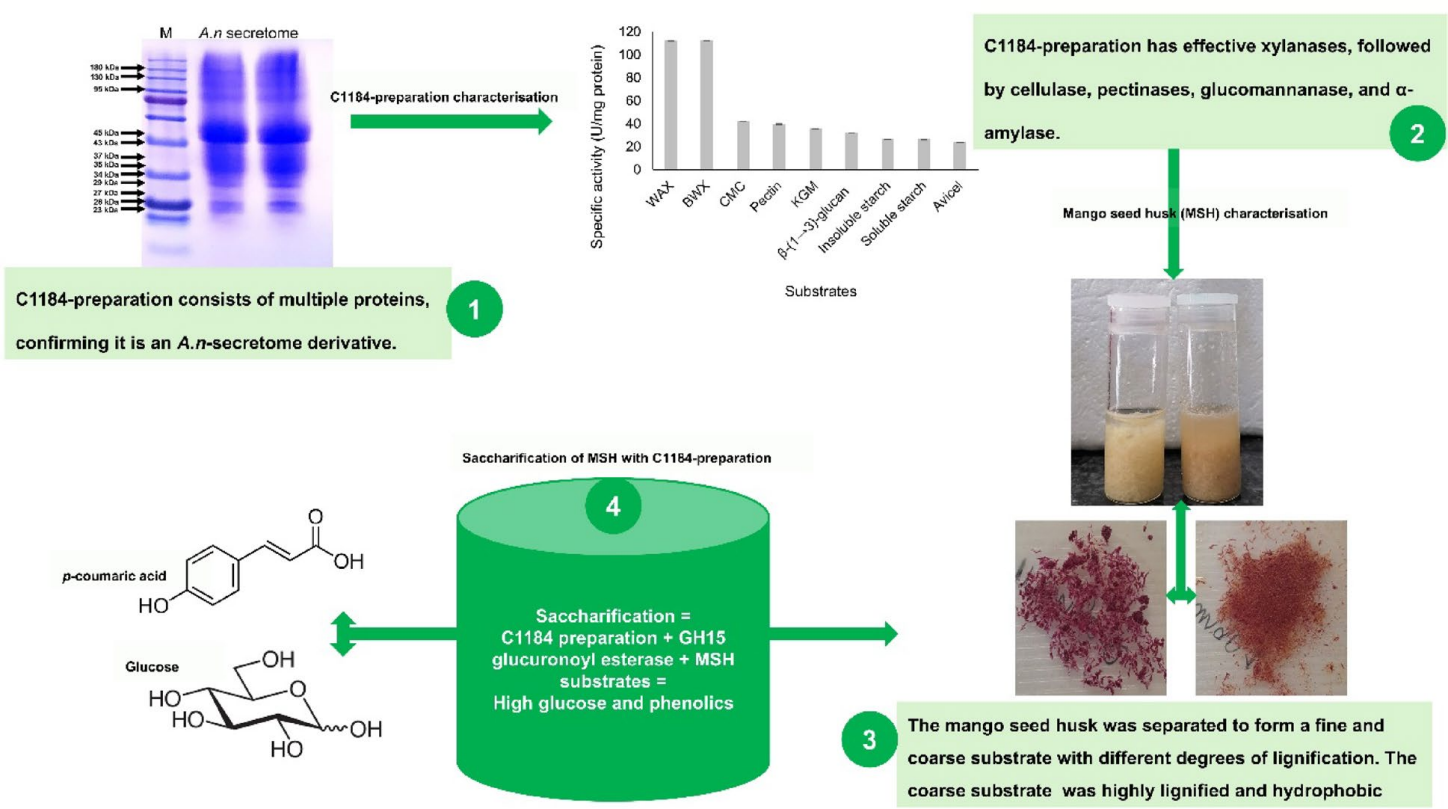

**Supplementary Information:**

The online version contains supplementary material available at 10.1186/s40643-025-00989-z.

## Introduction

Lignocellulosic waste biomass from agricultural processing is one of the essential sustainable resources for the production of biofuel and value-added products with climate and environment-friendly properties (Mujtaba et al. 2023). In the past decades, the agricultural by-products of harvesting monocotyledon crops such as wheat, barley, maize, rice and sugar cane have been reported in second-generation (2G) biorefineries (Balan et al. 2013; Chandel et al. 2018). These biomass resources are rich in the three major biopolymers, i.e. cellulose, hemicellulose and lignin, constituting about 30–45%, 15–40%, and 15–20% of the biomass dry weight, respectively (Janusz et al. 2017). Other agro-industrial residues, including waste tea residues, mango kernel and mango seed husk (MSH), which are less studied, hold significant potential for biorefinery applications in regions where they are more abundant, such as parts of Africa and Asia (Andrade et al. 2016; Bello and Chimphango 2021; Debnath et al. 2021; Manhongo et al. 2021; Mohotloane et al. 2023, 2024). Concerning the production of value-added products from mango by-products, most research has been focused on the peel and kernel fractions (Kittiphoom 2012), in part due to the high carbohydrate and nutritional content of the kernel (Choudhary et al. 2023; Yadav and Paudel 2022), while the seed husk (MSH) itself is an emerging renewable biomass source. Over the last decade, a few studies have reported on the use of MSH for the production of high-purity lignin and cellulose-rich pulp (Bello and Chimphango 2021), cellulose nanocrystals (Henrique et al. 2013), adsorbent for water treatment (Elizalde-González and Hernández-Montoya 2007) or bio-oil and furfural (Andrade et al. 2016). Currently, there has been limited focus on the conversion of MSH to fermentable soluble sugars through enzymatic saccharification (Siacor et al. 2021a, b). Recognizing that efficient conversion to such platform sugars can unlock new valorization pathways for MSH, we evaluated the potential of both untreated and pretreated MSH as sources of platform sugars. For benchmarking purposes, we utilised a commercial C1184 cellulase cocktail from *Aspergillus niger* marketed by Sigma-Aldrich as biocatalysts.

In general, MSH contains 39–56% (w/w) cellulose, 16–21% (w/w) hemicellulose and 12–26% (w/w) lignin, the exact composition depending on the type and origin of the mango and the preparation of the husk (Andrade et al. 2016; Elizalde-González and Hernández-Montoya 2007; Siacor et al. 2021a, b). While limited information is available on the carbohydrate composition of MSH (Chen et al. 2012), the main hemicellulosic polysaccharides in mango seed are xyloglucan and xylan (Bello and Chimphango 2022). In plant biomass, including MSH, lignin hinders enzymatic saccharification of biomass sterically, by covering the holocellulose (Mafa et al. 2020b; Van Dyk and Pletschke 2012) and by forming cross-links with hemicellulose through ester bonds (Mnich et al. 2020; Østby et al. 2020), and physico-chemically, by binding cellulases or hemicellulases non-productively. Hence, lignin removal from the biomass through chemical or enzymatic methods is highly beneficial to achieve greater saccharification of the holocellulose and to produce more soluble sugars such as glucose, xylose, and galactose. Therefore, Bello and Chimphango (2021) have developed an organosolv method, removing pure lignin from MSH and resulting in cellulose-rich fibres with more than 70% (w/w) cellulose and about 10% (w/w) hemicellulose.

In addition to lignin removal, different enzyme cocktail formulation strategies have been developed to effectively convert the polysaccharide fraction of lignocellulose to soluble fermentable sugars. Complete conversion of this biomass requires high enzyme loading per g of biomass. Due to the required high enzyme load, commercial cellulolytic enzymes are most often produced using genetically engineered *Trichoderma reesei* strains expressing recombinant enzymes, which are able to produce titres of extracellular protein over 100 g/L (Bischof et al. 2016). Alternatively, *Aspergillus* sp. (de Vries 2003) and *Thermothelomyces thermophilus* (previously *Myceliophthora thermophila*) (Visser et al. 2011) are in use by commercial enzyme producers. In addition to high enzyme loading, the broad diversity of carbohydrate-active enzymes (CAZymes) with complementing activities in the fungal secretomes is beneficial, and often necessary, for complete saccharification of various lignocellulosic feedstocks. For a better overview, CAZymes have been classified into classes, including glycoside hydrolases (GHs), carbohydrate esterases (CEs) and auxiliary activities (AAs), in the Carbohydrate-Active Enzymes database (http://www.cazy.org/), based on activity and sequence similarity (Drula et al. 2022). Notably, *T. reesei*, *Aspergillus* sp. and *T. thermophilus* differ in their repertoire of cellulolytic and hemicellulolytic enzymes (Berka et al. 2011; Martinez et al. 2008; Pel et al. 2007). While all these species contain similar numbers of cellobiohydrolases acting at the reducing (Cel7A) or non-reducing (Cel6A) end, endoglucanases (Cel7B and Cel5A) and β-glucosidases (Cel1A and Cel3B), *T. reesei* has two, *A. niger* has seven and *T. thermophilus* has twenty three AA9 lytic polysaccharide monooxygenases (LPMOs) for the oxidative depolymerisation of cellulose and other polysaccharides. Notably, endoglucanases and LPMOs are often promiscuous, being able to cleave cellulose and other plant polysaccharides (Østby et al. 2020). Furthermore, according to Borin et al. (2015), *A. niger* contains a complete xylanolytic enzyme cocktail including the GH10 and GH11 xylanases that cleave the main backbone of the xylan substrates; β-xylosidase, which further catalyses the xylanase products into xylose; and accessory enzymes, i.e., acetylesterase (CE1 and CE16), α-L-arabinofuranosidase (GH51, GH54 and GH62) and α-glucuronidase (GH67), which remove substitutions from the xylan backbone. Thus, an unpurified (i.e., secretome) or a partially purified cellulase preparation produced by these fungal strains often contains multiple enzymes and functions as an enzyme cocktail during saccharification of lignocellulosic biomass.

Glucuronoyl (GE) and hydroxycinnamoyl (including feruloyl, FAE) esterases play an important role in increasing enzymatic accessibility to plant cell wall polysaccharides (Puchart and Biely 2023). On the one hand, glucuronoyl esterases hydrolyse ester bonds formed between the free carboxyl of 4-*O*-methylated or non-methylated glucuronyl substitutions on glucuronoxylan and the aliphatic hydroxyl groups in lignin, thus separating the carbohydrate and lignin fractions more precisely than α-glucuronidases (Puchart and Biely 2023; Larsbrink and Lo Leggio 2023). On the other hand, hydroxycinnamoyl esterases remove hydroxycinnamoyl (mainly feruloyl and *p*-coumaroyl) substitutions from arabinoxylan. Some of these groups participate in cross-link formation between arabinoxylan and lignin, or, in the case of the feruloyl groups, between two feruloylated arabinoxylan chains via diferulic bridges (Dilokpimol et al. 2016; Puchart and Biely 2023). Notably, the genome of *A. niger* encodes over ten genes annotated as feruloyl esterases and no CE15 glucuronoyl esterase but a GH67 α-glucuronidase (Pel et al. 2007).

In this study, we assessed MSH, an underutilised renewable waste biomass, for the production of glucose and natural phenolic compounds through enzymatic processes. We focused on the role of lignin–carbohydrate linkages, specifically feruloyl and glucuronoyl esters, in biomass recalcitrance and their hydrolysis to enhance glucose release and phenolic compound extraction. We report the initial characterisation of the commercial cellulase from *A. niger* (product code C1184 by Merck, Sigma Aldrich), which revealed it as a cellulase cocktail with a range of CAZyme activities rather than a monocomponent enzyme. We then assessed the potential of the C1184 preparation for the valorization of MSH to produce glucose and phenolic compounds, both alone and in combination with glucuronoyl or feruloyl esterase. Our results highlight the importance of cleaving lignin–carbohydrate linkages formed via glucuronoyl esters for MSH valorization.

## Methods

### Materials

The commercial cellulase from *Aspergillus niger* (product code C1184) and β-glucosidase (Novozym 188) were purchased from Sigma-Aldrich (Johannesburg, South Africa). Avicel® PH-101 (prepared from wood; CAS No. 9004-34-6), pure pectin from apple (degree of esterification 50–75%; CAS No. 9000-69-5), carboxymethylcellulose [CMC; average molecular mass ~ 700,000 g/mol, medium viscosity; CAS No. 9004-32-4], soluble starch (CAS No. 9005-84-9), β-(1 → 3)-glucan from the algae *Euglena gracilis* (CAS No. 9051-97-2) and cellobiose (molecular mass: 342.30 g/mol; purity > 99.0%; CAS No. 528-50-7) were purchased from Sigma-Aldrich (Johannesburg, South Africa). The insoluble starch (Standardised regular maize starch control from the Total Starch Hexokinase Assay Kit; SKU: 700004350), red starch (soluble, partially depolymerised starch dyed with Procion Red MX-5B; SKU: 700,005,085), konjac glucomannan [KGM; monosaccharides ratio Mannose:Glucose = 60:40; low viscosity; purity > 98%; SKU: 700005012], azo-carob galactomannan (SKU: 700005040), mixed-linkage β-d-(1 → 3),(1 → 4)-glucan (MLG) from barley flour (molecular mass: 179,000 g/mol; low viscosity; purity ~ 95%; SKU: 700005000), mixed-linkage β-d-(1 → 3),(1 → 6)-glucan from fungal cell wall [Control yeast β-glucan preparation from the β-glucan Assay Kit (Yeast and Mushroom); SKU: 700004358], wheat arabinoxylan [WAX; monosaccharides ratio Arabinose:Xylose = 38:62; purity > 95%; SKU: 700005026; CAS No. 9040-27-1] and purified 4-*O*-methylglucuronoxylan from beechwood [BWX; monosaccharides ratio Xylose:Glucuronic Acid:Other sugars = 84:10.3:5.7; purity > 95%; SKU: 700005029; CAS No. 9014-63-5] were purchased from Megazyme (Bray, Ireland). Laminari-oligosaccharides (LOS) laminaribiose (molecular mass: 342.3 g/mol; purity > 95%; SKU: 700004959), laminaritriose (molecular mass: 504.4 g/mol; purity > 95%; SKU: 700004960), laminaritetraose (molecular mass: 666.6 g/mol; purity > 95%; SKU: 700004961) and laminaripentaose (molecular mass: 828.7 g/mol; purity > 95%; SKU: 700004962), cello-oligosaccharides (COS) cellotriose (molecular mass: 504.4 g/mol; purity > 95%; SKU: 700004949), cellotetraose (molecular mass: 666.6 g/mol; purity > 90%; SKU: 700004948) and cellopentaose (molecular mass: 828.7 g/mol; purity > 95%; SKU: 700004947), xylo-oligosaccharides (XOS) xylobiose (molecular mass: 282.2 g/mol; purity > 95%; SKU: 700004991), xylotriose (molecular mass: 414.4 g/mol; purity > 90%; SKU: 700004995), xylotetraose (molecular mass: 546.5 g/mol; purity > 95%; SKU: 700004994) and xylopentaose (molecular mass: 678.6 g/mol; purity > 95%; SKU: 700004993) as well as manno-oligosaccharides (MOS) mannobiose (molecular mass: 342.3 g/mol; purity > 95%; SKU: 700004972), mannotriose (molecular mass: 504.4 g/mol; purity > 95%; SKU: 700004976), mannotetraose (molecular mass: 666.6 g/mol; purity > 95%; SKU: 700004975) and mannopentaose (molecular mass: 828.7 g/mol; purity > 95%; SKU: 700004974) were purchased from Megazyme (Bray, Ireland). Furthermore, the CE1 feruloyl esterases (EC 3.1.1.73) *Ct*FAE_XynZ from *Clostridium thermocellum* (shortly *Ct*FAE; UniProt ID, P10478; product code E-FAEZCT) and *rm*FAE1 from a rumen microorganism (shortly *rm*FAE; product code E-FAERU) and the CE15 glucuronoyl esterase (EC 3.1.1.B11) *Rf*CE15 from *Ruminococcus flavefaciens* (shortly *Rf*GE; UniPort ID, Q9RLB8; product code E-GERF) were purchased from Megazyme (Bray, Ireland). Thin layer chromatography and all the analytical chemicals used in the study were purchased from Sigma (Johannesburg, South Africa), unless stated otherwise.

#### Preparation of the mango substrates

Mango fruit was purchased from the local fruit store in Bloemfontein South Africa during the summer of 2022. The mango seed husk (MSH) was separated from the fruit and kernel and then washed with distilled water at room temperature until only the fibres remained. The MSH was dried completely for seven days at 60 °C and ground to a fine powder with a coffee bean grinder (model AHCG01, produced by @Home, South Africa). The ground biomass was sieved and separated into two fractions: a fine powder with a particle size of < 2 mm (referred to as fine MSH) and coarse particles (> 2 mm) that could not go through the sieve. These MSH fractions were kept in an airtight container at room temperature until further analysis.

Previous studies have demonstrated that alkali pretreatment removes a substantial fraction of the hemicellulose and lignin contents from the biomass, thus facilitating the depolymerisation of cellulose (Government et al. 2019; Mafa et al. 2020b). Thus, the fine and coarse fractions of MSH (5 g dry weight each) were pretreated with 20% (w/v) NaOH (100 mL NaOH per g biomass) for 6 h at 60 °C to produce alkali-pretreated MSH. The samples were centrifuged at 5000*g*, and the pellet was washed 6 times with 1L distilled water until the filtrate was clear. After washing, the pH was adjusted from pH 10 to 6 with 2 M HCl, followed by centrifuging the samples at 5000*g* at 4 °C for 30 min. The pellets were dried at 60 °C for 72 h and then stored in an airtight container before use.

### Analysis of proteins in the C1184 preparation

The powdered cellulase from *A. niger* produced by Sigma (referred to as C1184 preparation in the subsequent sections) was weighed and dissolved in 50 mM sodium citrate buffer (pH 5.0). The composition of the C1184 preparation was determined using 12% (w/v) sodium dodecyl sulfate–polyacrylamide gel electrophoresis (SDS-PAGE), according to Laemmli (1970). The total protein concentration was measured using the Bradford method (1976). To be able to analyse the C1184 preparation with SDS-PAGE and the Bradford method, we had to prepare a 100 mg/mL stock, which was equivalent to 1 mg/mL total protein, as determined with the Bradford method. It is important to note that some of the chemicals used to stabilise the C1184 preparation during lyophilisation used by the manufacturer (Sigma-Aldrich/Merck) lead to overestimation of the enzyme/protein concentration. Therefore, in the assays below, an enzyme stock made up of 1 mg/mL protein [equivalent to 100 mg powder dissolved in 1 mL 50 mM sodium citrate buffer (pH 5.0)] was used.

### Substrate specificity assays of the C1184 preparation

We assessed the activity of the C1184 preparation on cellulose (CMC and Avicel), xylan (WAX and BWX), β-glucan [algal β-(1 → 3)-glucan, barley MLG and fungal β-(1 → 3),(1 → 6)-glucan], mannan (KGM and azo-carob galactomannan), starch (red starch) and pectin from apple. C1184, at about 0.125 mg/mL final protein concentration, was used to treat 1% (w/v) substrate suspended/dissolved in 50 mM sodium citrate buffer (pH 5.0). The reactions for the soluble substrates were performed by incubating the reaction tubes in the digital dry-heating block (Model Digital E-DB120-D, United Scientific PTY LTD, Cape Town, South Africa) set at 40 °C for 1 h. For Avicel, reaction tubes were incubated at 40 °C for 5 h in the same heating block, with vortexing every hour. The total reducing sugars produced in the reactions by the C1184 preparation were measured using 3,5-dinitrosalicylic acid (DNS) based on the Miller method (1959) with modification described earlier in by Mafa et al. (2020a, b). The samples were measured at 540 nm using a GENESYS™ 180 UV–Vis spectrophotometer (North York, ON, Canada), and glucose or xylose was used as standard. For V_max_ the specific activity was calculated by dividing the concentration of the formed reducing ends (in µmol/mL) with the effective protein concentration in the reaction (in mg protein/mL) and the reaction time (in min).

### In-gel activity assay for cellulolytic, xylanolytic and mannanolytic enzymes in the C1184 preparation

Zymograms were performed using SDS-PAGE, each containing 0.1% (w/v) of CMC, WAX, red starch or azo-carob galactomannan substrate, to determine cellulolytic, xylanolytic, amylolytic or mannanolytic enzyme activities in the C1184 preparation, respectively. For each zymogram, 10 µL of 1 mg/mL enzyme stock (see section "Analysis of proteins in the C1184 preparation" for details) was mixed with 40 µL sample buffer [0.2 M Tris–HCl pH 6.8, 10% (w/v) SDS, 20% v/v glycerol and 0.05% (w/v) Bromophenol Blue] to give an enzyme solution with 0.2 mg/mL protein concentration. Of this, 15 µL was loaded on a 12% SDS-PAGE gel containing either of the substrates and run for 2 h at 118 V. After separation, the gels were renatured in 10 mM Tris–HCl buffer (pH 7.5) containing 1% (v/v) Triton-X-100 for 20 min at room temperature. After renaturation, the enzymatic activity was initiated by incubating the gel in 50 mM sodium citrate buffer (pH 5.0) for 30 min at 37 °C in a BUCHI B-350 water bath (BÜCHI Labortechnik AG, Flawil, Switzerland). The gels containing CMC or WAX were stained for 10 min using 0.05% (w/v) Congo red and subsequently destained using 1 M NaCl until a clear band appeared. For the dyed substrates, staining was unnecessary as the enzyme activity led to the development of clear bands. The Color Prestained Protein Standard (Broad Range, 10–250 kDa) from New England Biolabs (Ipswich, MA, USA) was used to determine the molecular mass of the proteins showing activity, which were viewed as clear bands.

### Determination of pH and temperature optima, thermostability, and the apparent kinetics of cellulolytic, xylanolytic and mannanolytic activities in the C1184 preparation

Given that the C1184 preparation displayed activity towards cellulose, xylan and glucomannan (but not galactomannan), we analysed these activities in detail using CMC, WAX and KGM. To determine the pH and temperature optima and thermostability, enzyme reactions were set up with 1% (w/v) substrate and 0.125 mg/mL (protein basis) C1184 preparation [diluted from a 1 mg (protein)/mL enzyme stock, see above]. The pH optima were assayed at 37 °C for 60 min using the following buffer systems: 50 mM sodium citrate buffer at pH 4.0 or 5.0, 50 mM sodium phosphate buffer at pH 6.0 or 7.0 and 50 mM Tris–HCl at pH 8.0 or 9.0. Temperature optima were assayed in 50 mM sodium citrate buffer (pH 4.0) for 60 min using temperatures between 20–80 °C, while the thermostability assays were performed in 50 mM sodium citrate buffer (pH 4.0) for 60 min at 37 °C after pre-incubating the secretome at 37, 50 and 70 °C for 1, 5 and 24 h. The DNS reagent was used to measure the total reducing sugars, according to Miller (1959) with modification as described in section "Substrate specificity assays of the C1184 preparation". The activities are reported as relative activities, normalized to the condition with the highest activity (i.e., the greatest formation of reducing sugars) was observed. For apparent kinetics studies, CMC, WAX and KGM substrate concentrations were varied from 1.0 to 14 mg/mL dissolved in 50 mM sodium citrate buffer (pH 4.0). The reaction was initiated by adding 0.125 mg/mL (protein basis) C1184 preparation, followed by the incubation of the reaction at 50 °C for 1 h. The kinetic constants, K_m_ and V_max_, were calculated from the Michaelis–Menten equation, autocorrected using the solver in Microsoft Excel (version 2016) and validated with calculations of KaleidaGraph (version 4.5, Synergy Software, Reading, PA, USA). It is essential to note that the apparent kinetic constants were used for comparative analysis and to validate the differences in the performance of xylanases, cellulases and mannanases.

### Assessing the ability of the C1184 preparation to depolymerise oligosaccharides into monomers

Oligosaccharides with COS, XOS, MOS and LOS with degrees of polymerisation (DPs) 2–5 (1 mg/mL) were incubated with 0.12 mg/mL (protein basis) pre-dissolved C1184 preparation in 50 mL sodium citrate buffer (pH 4.0) for 1 h at 37 °C in a digital dry heating block (Model Digital E-DB120-D, United Scientific PTY LTD, South Africa) without mixing. The reaction was terminated by incubation at 95 °C for 5 min, and then samples were allowed to cool. Of the hydrolysate, 3 µL was spotted three times on thin-layer chromatography (TLC) plates (Silica Gel 60G F254 HPTLC plates from Merck, Darmstadt, Germany), and the products were separated with a mobile phase containing *n*-butanol:acetic acid:water (2:1:1, v/v/v). Mixtures of the unhydrolysed oligosaccharides were used as standards. Carbohydrates were detected by briefly submerging the plates in ethanol containing 5% (v/v) sulphuric acid and 0.3% (w/v) α-naphthol (Molisch's stain). Plates were then air-dried and subsequently heated at 120 °C for 10 min.

### Characterisation of mango seed Husk (MSH)

#### Surface analysis of MSH

The morphology and topological structure of the fine and coarse fractions of MSH were analysed by scanning electron microscopy (SEM) as described by Mohotloane et al. (2024). In addition, the elemental analysis was performed on an SEM FEG Quanta 450 (Joel, Tokyo, Japan) with energy-dispersive X-ray spectroscopy (EDS). The samples were deposited on carbon tape and metalised with metallic silver by a Quorum QT150ES Metallizer (QUORUM, Kent, UK). A pressure of 10 Pa was applied to the SEM chamber, along with an incident electron beam of 20 kV.

We used the Wiesner reaction to determine whether lignin is present in both the fine and coarse fractions MSH. The reaction was performed by pouring 500 µL of 1% (w/v) phloroglucinol dissolved in absolute ethanol on 300 mg MSH samples, then 35% HCl was added by pipetting 20 µL in a dropwise manner and mixed. The colour was allowed to develop for 10 min.

#### Determining crystallinity of MSH

The degree of crystallinity was determined using X-ray diffraction (XRD), according to Park et al. (2010). A Bruker-AXS D8 Discover diffractometer (Bruker, Madison, WI, USA) was used to perform XRD analysis using Cu Kα radiation at 40 kV and 130 mA at a Coupled 2θ/ω scanning angle and a speed of 0.5°/min. Peak height was used to calculate the crystallinity index as described by Park et al. (2010).

### Saccharification of MSH with the C1184 preparation supplemented with feruloyl or glucuronoyl esterase

Feruloyl and glucuronoyl esterases improve the hydrolysis of the lignocellulosic biomass by removing ester bonds that crosslink lignin and hemicellulose. To determine the effects of cleaving such ester bonds, the fine and coarse fractions of MSH (after grinding but without additional pretreatment) were suspended at 1% (w/v) final concentration in 50 mM sodium citrate buffer (pH 5.0), and subjected to enzyme treatment with 0.25 mg/mL C1184 alone or 0.125 mg/mL C1184 preparation supplemented with 0.125 mg/mL *Ct*FAE, *rm*FAE or *Rf*GE, corresponding to an enzyme loading of 25 mg protein/g dry biomass. The reactions (500 µL total volume) were incubated at 40 °C for 24 h in a digital dry heating block (Model Digital E-DB120-D, United Scientific PTY LTD, South Africa), with mixing the samples every 2 h for the first 8 h and the last 8 h of the reaction. The glucose release was determined with the glucose assay kit (GOPOD kit) from Megazyme; and the dissolved aromatic compounds, also referred to as solubilised lignin, were measured according to Ainsworth and Gillespie (2007).

### Saccharification of alkali-pretreated MSH with the C1184 preparation supplemented with β-glucosidase

Alkali-pretreated MSH fractions were suspended in 50 mM sodium citrate buffer (pH 5.0) at 1% (w/v), and the reaction was initiated by adding 0.5, 1, 3 or 5 mg/mL enzymes, containing the C1184 preparation (90%, w/w) supplemented with 10% (w/w) β-glucosidase (Novozym 188). The enzyme loadings corresponded to 5–50 mg protein/g dry biomass. The reaction was performed at 40 °C for 24 h, with mixing the samples every 2 h for the first 8 h and the last 8 h of the reaction, using a digital dry heating block (Model Digital E-DB120-D, United Scientific PTY LTD, South Africa) for 24 h. The glucose release was quantified using the glucose assay kit (GOPOD kit) from Megazyme and expressed as mg glucose in g of dry-weight biomass.

## Results and discussion

### Substrate specificity of the C1184 preparation from A. niger

**Fig. 1:**
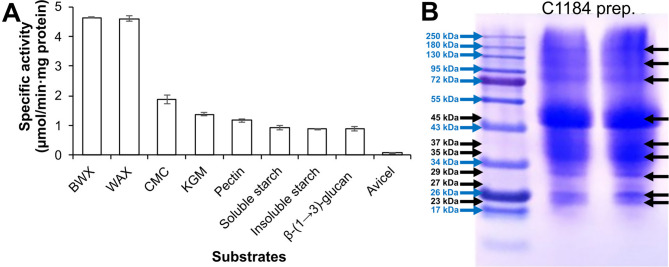
Enzyme activity screening and protein analysis of the C1184 cellulase preparation from *A. niger*. **A** Enzyme activity screening. Reactions were set up with 1% (w/v) beechwood glucuronoxylan (BWX), wheat arabinoxylan (WAX), carboxymethylcellulose (CMC), konjac glucomannan (KGM), pectin from apple, soluble starch, insoluble starch, algal β-(1 → 3)-glucan or Avicel as substrate and 0.125 mg/mL C1184 (total protein level) in 50 mM sodium citrate buffer (pH 5.0) and incubated at 40 °C for 1 h, or for 5 h with Avicel. The experiments were performed in triplicate; the values represent the means, and the error bars represent the standard deviation. **B** Protein analysis. The SDS-PAGE, performed in duplicate, clearly shows the major protein bands in the *A. niger* secretome. Lane M, Colour Prestained Protein Standard (Broad Range, 10–250 kDa) marker. Blue arrows indicate the molecular mass in the Protein Standard; additional black arrows on the left indicate the molecular mass of additional protein bands appearing in C1184; black arrows on the right mark the main protein bands detected in C1184

The manufacturer indicated that the cellulase from *A. niger* (purchased from Sigma; product code C1184) is active on polymeric β-d-glucan substrates with β-(1 → 4)-linkages or mixed β-(1 → 3) and β-(1 → 4) linkages and on cello-oligosaccharides with a DP of 3–6. Furthermore, this product is devoid of β-glucosidase activity, while it can cleave glycosaminoglycan chains off a core protein that is attached to a serine via an xylosyl unit. The suggested reaction conditions for the enzyme preparation were specified as pH 5.0 and 37 °C (https://www.sigmaaldrich.com/NO/en/product/sigma/c1184). To better understand the potential of the C1184 preparation from *A. niger* for the saccharification of MSH, we assessed its substrate specificity on a broad range of plant cell wall polysaccharides (Fig. 1A). The results show that the C1184 preparation had significantly higher activity on xylan substrates [wheat arabinoxylan (WAX) and beechwood glucuronoxylan (BWX)] than cellulose (Avicel and CMC). Furthermore, the C1184 preparation displayed activity on pectin from apple, konjac glucomannan (KGM), algal β-(1 → 3)-glucan as well as insoluble and soluble starch. Activities towards CMC, WAX, BWX and KGM by cellulolytic, xylanolytic and mannanolytic enzyme activities, respectively, were confirmed using TLC (Figure S1).

While substrate promiscuity is not uncommon among cellulases, such cellulases show the ability to cleave various polysaccharides with β-(1 → 4)-linked sugar units in the polysaccharide backbone, including cellulose, xyloglucan, mixed-linkage β-glucan, glucomannan and xylan (Mafa et al. 2021; Payne et al. 2015; Vlasenko et al. 2010). The fact that the C1184 preparation displayed activities towards β-(1 → 3)-glucan, pectin and starch, activities that have not been attributed to secreted fungal endoglucanases before, at levels that were comparable to the activity on cellulose (Fig. 1A) indicates that the C1184 preparation from *A. niger* is rather a derivative of a fungal secretome than a multifunctional endoglucanase.

The SDS-PAGE analysis further indicated that the C1184 preparation from *A. niger* contains a major protein of approx. 45 kDa band and a number of proteins with different molecular masses (Fig. 1B), corroborating that the preparation may rather be a cellulase cocktail derived from an *A. niger* secretome than a pure endo-β-(1 → 4)-glucanase. Notably, *A. niger* species encode more than 450 CAZymes (Aguilar-Pontes et al. 2018; Arnaud et al. 2012; Pel et al. 2007), which include several cellulases, xylanases, mannanases and pectinases. Previously, Wang et al. (2018) have used functional zymography to demonstrate the occurrence and abundance of functional endoglucanases and endo-xylanases in *A. fumigatus* secretomes obtained under various growth conditions. We decided to follow a similar approach to further characterise the C1184 preparation in the next section.

### Zymogram analysis of the C1184 preparation from *A. niger*

**Fig. 2:**
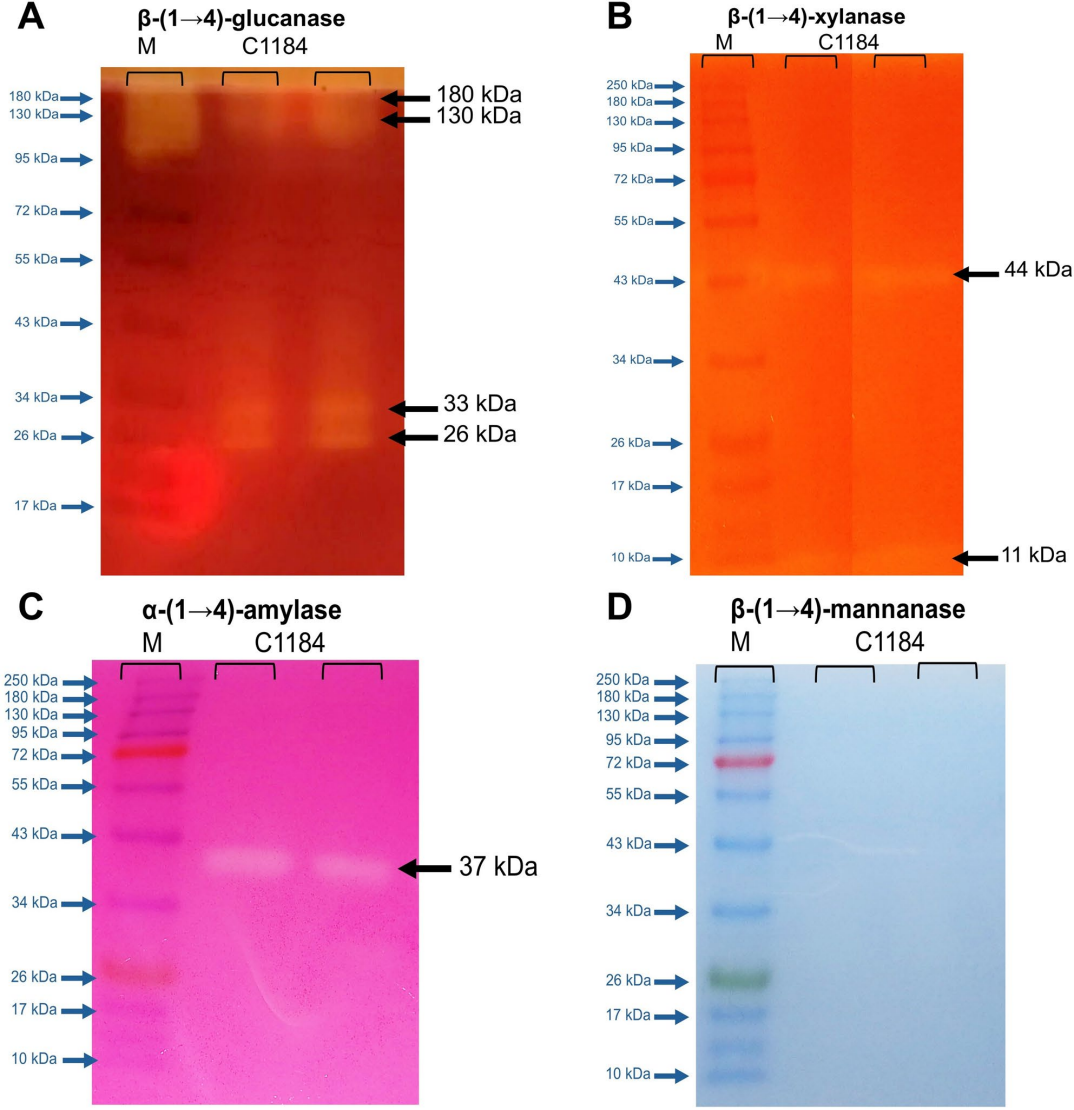
Zymogram analysis of the C1184 preparation for cellulolytic, xylanolytic, mannanolytic and amylolytic activities. **A** Endo-β-(1 → 4)-glucanase activity using carboxymethylcellulose (CMC), **B** endo-β-(1 → 4)-xylanase activity using wheat arabinoxylan (WAX), **C** α-(1 → 4)-amylase activity using red starch and **D** endo-β-(1 → 4)-mannanase activity using azo-carob galactomannan. The gels contained 0.1% (w/v) substrate, and 3 µg protein of C1184 was loaded on each gel. Blue arrows on the left indicate the molecular mass in the marker (Color Prestained Protein Standard; Broad Range, 10–250 kDa); black triangles on the left and black arrows on the right indicate the detected protein bands with activity in C1184B

Zymogram analysis was used to validate the cellulolytic, mannanolytic, xylanolytic and amylolytic activities detected in the C1184 preparation and to identify the molecular masses of the proteins with corresponding activities. The differential enzyme activity profiles were expected to reveal which of the previously detected activities correspond to the major protein (with 45 kDa) and which additional proteins from the expression host (*Aspergillus niger*) contribute to cellulase, β-(1 → 4)-xylanase, β-(1 → 4)-mannanase or α-amylase activities in the C1184 preparation. The zymogram set up with 0.1% (w/v) CMC revealed that the secretome had at least four different enzymes with endo-β- (1 → 4)-glucanase activity, displaying the following molecular masses: 26, 33, 130 and 180 kDa (Fig. 2A). These protein sizes correspond to those bands observed with SDS-PAGE analysis (Fig. 1B) at 26, 34, 130 and 180 kDa, respectively. No clearing zone at about 45 kDa, corresponding to the strongest protein band in the SDS-PAGE gel, was detected. The endoglucanase with 26 kDa likely corresponds to the 26-kDa endoglucanase isolated from *A. niger* by Hurst et al. (1977); and the endoglucanase with 33 kDa likely corresponds to the 31-kDa endoglucanase identified and purified from *A. niger* by Okada (1985). The other two proteins with molecular masses of 130 and 180 kDa are unusually large for endo-β-(1 → 4)-glucanases and more likely correspond to β-glucosidases (de Vries and Visser 2001). Considering that the C1184 preparation was not denatured prior to being subjected to zymography (as opposed to SDS-PAGE analysis), a potential explanation for the endoglucanase activity at 130 or 180 kDa could be that these bands may correspond to a multimodular protein. Notably, Ximenes et al. (1996) showed the presence of a multimodular β-glucosidase with cellulase function from *A. fumigatus*, with a molecular mass of 130 kDa, that dissociated into 90 kDa catalytically active and 45 kDa inactive subunits under denaturing conditions. In any case, future studies are needed to fully elucidate the nature of the proteins forming bands at 130 and 180 kDa.

On the other hand, the zymogram with 0.1% (w/v) WAX revealed the presence of two distinct bands of xylanolytic enzymes with molecular masses of 11 and 44 kDa (Fig. 2B). Previously, Frederick et al. (1985) purified and characterised two xylan-active isozymes with 13 kDa, Shei et al. (1985) characterised a 14 kDa xylanase, and Amir et al. (2013) purified 43 kDa xylanase from *A. niger* secretomes, all of which had molecular masses similar to those reported in this study. The facts that the C1184 cellulase preparation from *A. niger* gave the highest specific activity on xylan in the substrate specificity assay shown in Fig. 1A and that the major protein component had a molecular mass close to 45 kDa (Fig. 1B) further corroborate that the major protein in the C1184 preparation corresponds to xylanase.

The zymogram with 0.1% (w/v) red starch substrate further revealed α-amylase activity for a protein with a molecular mass of about 37 kDa (Fig. 2C). The zymogram with azo-carob galactomannan as a substrate, on the other hand, showed no activity (Fig. 2D) despite the presence of mannanases being secreted by *A. niger* (de Vries and Visser 2001) and the activity of the C1184 preparation on KGM observed above (Fig. 1A and Figure S1). Activity on KGM could be the result of the action of (promiscuous) endoglucanases and endo-β- (1 → 4)-mannanases, where the action of both enzyme types is hindered by galactosylations in carob galactomannan (McCleary and Matheson 1983). Indeed, endo-β-(1 → 4)-mannanases present in the C1184 preparation were able to cleave non-substituted mannooligosaccharides as shown further below (Fig. 4A).

### Biochemical characteristics and enzyme kinetics of cellulase, xylanase and mannanase activities in the C1184 preparation

**Fig. 3:**
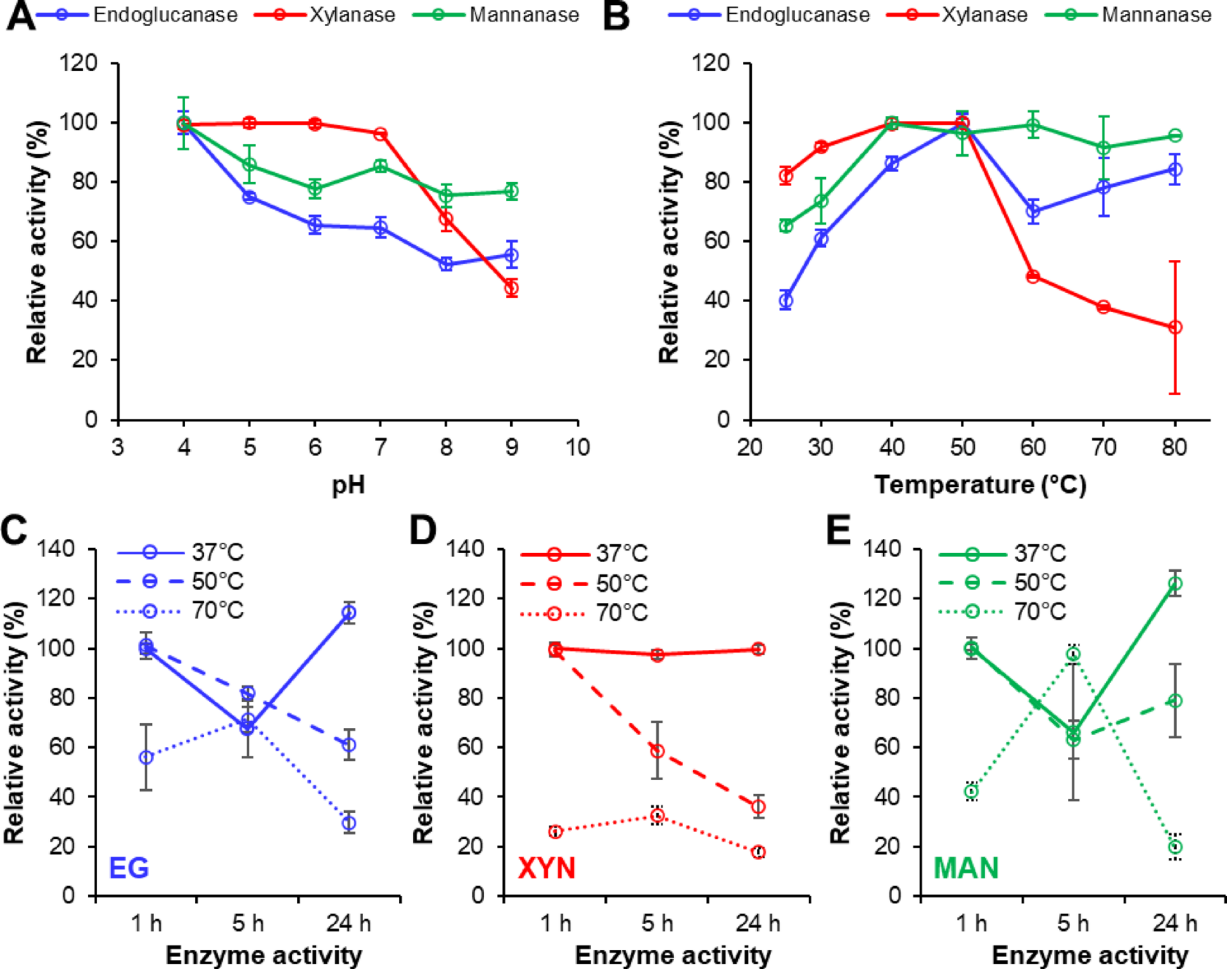
Characterisation of cellulolytic, xylanolytic and mannanolytic activities in the C1184 preparation. The pH (**A**) and temperature optima (**B**) and thermostability (**C**–**E**) of the cellulolytic (**A**–**C**), xylanolytic (**A**, **B**, **D**) and mannanolytic (**A**, **B**, **E**) enzymes in the C1184 preparation. Carboxymethylcellulose (CMC), wheat arabinoxylan (WAX) and konjac glucomannan (KGM) were used as substrates to assay overall endo-β-(1 → 4)-glucanase, endo-β-(1 → 4)-xylanase and endo-β-(1 → 4)-mannanase activities, respectively. Enzyme activities are expressed in relation to the activity levels after 1 h pre-incubation at 37 °C. The experiments were performed in triplicate; the values represent the means, and the error bars show the standard deviation

Preliminary studies of thermostability (conducted at pH 5.0, according to the manufacturer’s recommendation) indicated that the different enzyme activities, i.e., cellulase [or endo-β-(1 → 4)-glucanase], endo-β-(1 → 4)-xylanase, endo-β-(1 → 4)-mannanase, endo-β-(1 → 3)-glucanase and pectinase, in the C1184 preparation displayed various stabilities after pre-incubation of the enzyme preparation at 37 °C, 50 °C or 70 °C for up to 24 h (Figure S2). α-Amylases and pectinases were slightly less sensitive to being pre-incubated up to 5 h at elevated temperatures. Among these activities, we decided to focus on the detailed characterisation of the overall cellulolytic, xylanolytic and mannanolytic activities of the C1184 preparation because these activities are vital for the valorization of lignocellulosic waste biomass (Mafa and Malgas 2023; Mafa et al. 2020b; Malgas et al. 2019). Regarding the operational pH under incubation at 37 °C for 1 h, the overall endo-β-(1 → 4)-glucanase and endo-β-(1 → 4)-mannanase activities in the C1184 preparation displayed the highest activity at pH 4.0 (Fig. 3A). There was a gradual decrease in activity from 4.0 to 8.0, with activities at pH 5.0 reaching 75% and 86% of the highest activity (observed at pH 4.0), respectively. Interestingly, the endo-β-(1 → 4)-xylanase enzymes showed a broad range of pH optimum from pH 4.0–6.0 (> 99% of the highest activity recorded at pH 5.0), retaining 97% of the enzyme activity at pH 7.0. The xylanases lost significant enzyme activity under alkaline conditions, retaining only 44% of the enzyme activity at pH 9.0. At pH 9.0, the endo-β-(1 → 4)-glucanases and endo-β-(1 → 4)-mannanases retained 56% and 77% of the overall activity, respectively.

Next, we assessed the temperature optima of the C1184 preparation at pH 4.0 (Fig. 3B). After 1 h incubation, the endo-β-(1 → 4)-xylanase enzymes showed the highest activity (> 99%) at 40 and 50 °C, displaying only 31–48% of the highest activity at 60 °C and higher temperatures. In contrast, the endo-β-(1 → 4)-mannanases displayed temperature optimum at 40 °C and retained 96–99.5% of the relative activity from 50 to 80 °C, suggesting that the mannanases were significantly more thermotolerant than the xylanases in the C1184 preparation. Endo-β-(1 → 4)-glucanases showed optimum activity at 50 °C, retaining 70–85% relative enzyme activity between 60 and 80 °C. Importantly, the thermostability studies confirmed that neither of the enzymes suffered from thermal inactivation during the 1-h reactions performed at the optimal temperature as all enzyme types retained their overall activity after 1 h incubation at up to 50 °C prior to the reactions performed at 37 °C (Fig. 3C–E). In contrast, endoglucanases (Fig. 3C) and mannanases (Fig. 3E) retained 56% and 40% of their overall activity, respectively, while xylanases lost 74% of the overall activity after 1 h of pre-incubation at 70 °C (Fig. 3D). Notably, the endoglucanases and mannanases displayed a much lower relative activity than xylanases at lower temperatures (25 and 30 °C; see Fig. 3B). Taken together, these observations indicate that the C1184 preparation contains mesophilic xylanases and thermotolerant endoglucanases and mannanases, corroborating that the C1184 preparation from *A. niger* is rather a derivative of a fungal secretome than a multifunctional endoglucanase.

The kinetic studies of the overall cellulase, xylanase and mannanase activities in the C1184 preparation were performed at the pH and temperature optima of the preparation, i.e. at pH 4.0 and 50 °C, using 1.0 to 14 mg/mL CMC, WAX and KGM, respectively, as substrates. The findings show that the apparent V_max_ value for the overall xylanase activity was the highest, 1.8-fold higher than that of the cellulase and 4.1-fold higher than that of the mannanase activity. At the same time, the overall xylanase activity showed a lesser affinity towards WAX (K_m_ of 6.80 mg/mL) than the endoglucanase affinity towards CMC (2.49 mg/mL), with mannanases exhibiting the highest apparent affinity (K_m_ of 1.53 mg/mL) towards KGM (Table 1).

**Table 1:** Apparent kinetic parameters of the overall cellulolytic, xylanolytic and mannanolytic enzyme activities in the C1184 preparation

Activity	V_max_ (µmol mL^−1^ min^−1^)	K_m_ (mg mL^−1^)
Xylanase	4.284	6.80
Endoglucanase	2.397	2.49
Mannanase	1.055	1.53

The experiments were conducted in triplicates; carboxymethylcellulose, wheat arabinoxylan, and konjac glucomannan were used for assaying the kinetics of endoglucanase, xylanase and mannanase activities. It is essential to note that the apparent kinetic constants were used for comparative analysis and to validate the differences in the performance of xylanases, cellulases and mannanases in the C1184 preparation

Our findings are well in agreement with the literature data. Dobrev and Zhekova (2012) has purified an endoglucanase from the xylanase-producing *A. niger* B03 (molecular mass of about 27–29 kDa) with a pH optimum at 3–4, retaining more than 80% relative activity between pH 2.5–4.5. This enzyme retains 92% of its activity after 3.5 h pre-incubation at 40 °C and 60% of its activity after 45 min pre-incubation at 60 °C (Dobrev and Zhekova 2012). In another study, endoglucanases partially purified from *A. niger* ANL301 have shown multiple pH optima (at pH 3.5, 5.5 and 7.0) and a temperature optimum at 50 °C (Chinedu et al. 2011). Sulyman et al. (2020) have also shown that a 13.5 kDa endoglucanase purified from an *A. niger* secretome displays optimum activity at pH 4.0 and 40 °C. These studies support our findings that the overall endoglucanase activity in the C1184 preparation was the highest in the acidic pH range (at about pH 4.0) and at 50 °C, and that about 80% of the activity could be retained at 37 °C even after 24 h of incubation.

Regarding xylanases, Sherief (1990) has purified a 45-kDa endoxylanase with maximal activity at pH 5.0 and 55 °C. Furthermore, Amir et al*.* (Amir et al. 2013) have purified a 43-kDa xylanase from an isolate of *A. fumigatus* with a maximal activity at 30–40 °C up to 3 h of incubation, retaining its activity over a broad pH range (pH 4.0–10.0). Recently, Ravichandra et al. (2023) have also characterised a 43-kDa xylanase from *A. fumigatus* RSP-8 with optimal activity at pH 5.0 and 50 °C, with good stability up to 40–50 °C over 60 min. Furthermore, two xylanases with 13 kDa have been characterised by Frederick et al. (1985), showing pH optima of 5.0–6.0 and a temperature optimum of 45 °C. A third, 14-kDa xylanase purified from the same crude preparation (Shei et al. 1985) has optimum pH at 4.9 and displays the highest activity at 45 °C. Furthermore, a 35-kDa xylanase from *A. tamarii* BLU37 displays pH and temperature optima at 6.0 and 60 °C when using 30 min incubations (Monclaro et al. 2016). Another, 22-kDa xylanase from *A. tamarii* BLU37 displays a pH optimum of 5.5 and a temperature optimum of 50 °C (Monclaro et al. 2019). The latter xylanase rapidly loses activity between 65 and 80 °C, a trend also observed in the current study. Bhushan et al. (2015) have also demonstrated that, when using 15-min incubation, a 35-kDa xylanase from *A. flavus* MTCC 9390 shows an optimum pH of 5.0 and highest activity at 60 °C, which is followed by a rapid decrease in activity between 70 and 80 °C. On the other hand, Ding et al. (2018) have shown that a 32-kDa xylanase has an optimal pH 6.0 and temperature 50 °C when incubating the reactions for 10 min. Notably, within 60 min, this xylanase loses about 70% of its activity at 60 °C and about 25% of its relative activity at 40 °C within 60 min. These findings match the substantial enzyme inactivation seen above at 50 °C in our study when using 60-min incubations.

Regarding mannanases, Ademark et al. (1998) purified a 40-kDa β-mannanase from an *A. niger* culture broth with highest activity at pH 3.5 and stability over a broad pH range of 3.5–7.0. On the other hand, Yu et al. (2015) have expressed a thermostable 38-kDa β-mannanase from *A. niger* in *P. pastoris* and found that the enzyme retained 60% enzyme activity on locust bean gum after incubating for 2 h at 80 °C. Furthermore, a 48-kDa GH26 endo-1,4 β-mannanase from *A. niger*, marketed by Megazyme (product code E-BMANN), has a reported pH optimum 3.9 and temperature optimum at 60 °C, and it is recommended to be used at pH 4.0 and up to 40 °C, which are in line with our data.

Importantly, the overall xylanase, endoglucanase and mannanase activities in the C1184 preparation retained 100% activity at 37 °C after incubation for more than 24 h at pH 4.0, indicating that the C1184 preparation is suitable for biomass degradation studies at 37 °C over longer periods. Of note, our activity data indicate that these activities are 1–2 orders of magnitude lower than those present in commercial enzyme preparations. Additionally, the optimal pH (4.0) and temperature (37 °C) are somewhat lower than the pH (4.0–5.0) and temperature (50–60 °C) recommended for the most common *T. reesei* and *A. niger*-based cellulase preparations (Chylenski et al. 2017; Kabel et al. 2006; Nieves et al. 1998; Yang et al. 2017).

### Oligosaccharides hydrolysis patterns produced by the C1184 preparation

**Fig. 4:**
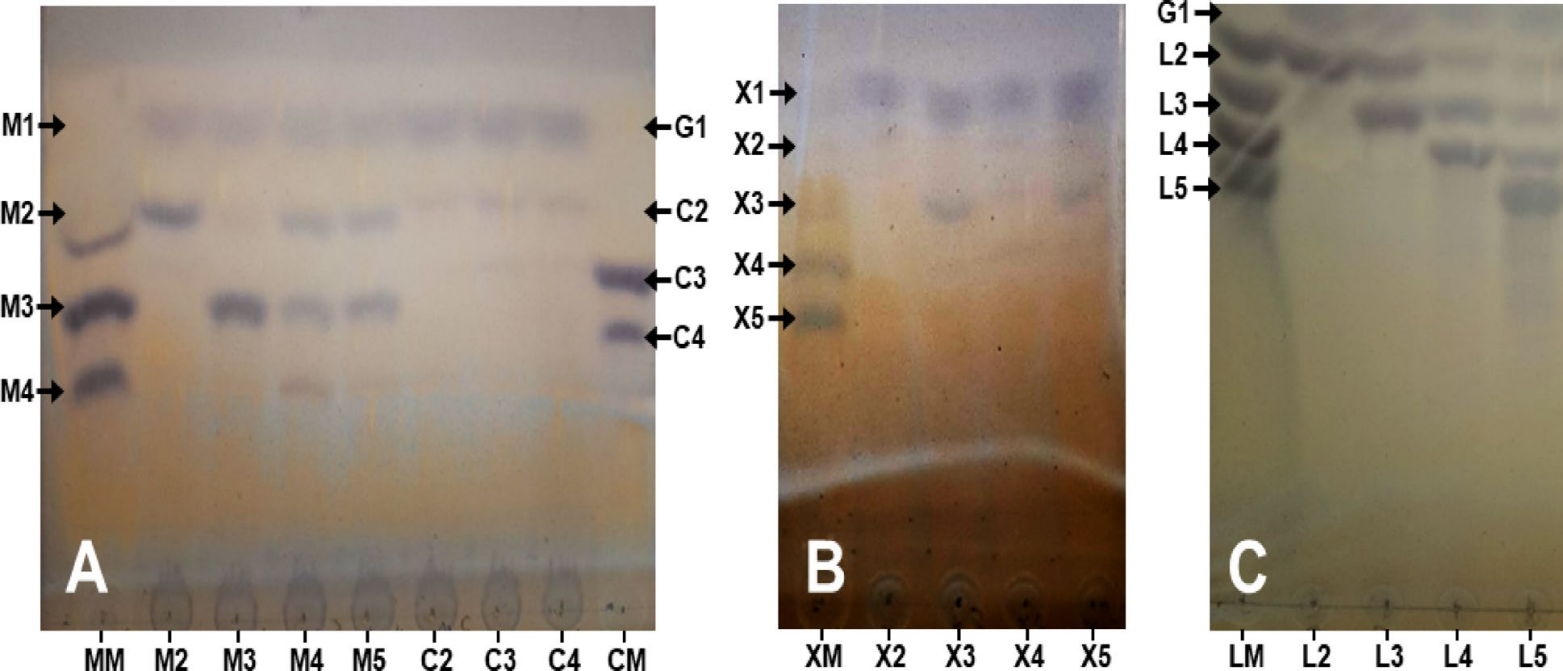
Thin-layer chromatography of the products generated by the C1184 preparation from oligosaccharides. The figures demonstrate depolymerisation of **A** manno-oligosaccharides (MOS) and cello-oligosaccharides (COS), **B** xylo-oligosaccharides (XOS) and **C** laminari-oligosaccharides (LOS) by the C1184 preparation. The lanes with markers are annotated as follows: MM, MOS with DP 2–4; CM, COS with DP 3–4; XM, XOS with DP 3–5; LM, LOS with DP 2–5. The lanes with hydrolysis products are annotated by the substrate used in the reaction, i.e.: M2–M5, MOS with DP 2–5; C2–C4, COS with DP 2–4; X2–X5 XOS with DP 2–5; L2–L5, LOS with DP 2–5, respectively. The formed oligosaccharides are annotated accordingly as M2–M4, C2–C4, X2–X5 and L2–L5, respectively. The formed sugar monomers are annotated as follows: M1, mannose; G1, glucose; X1, xylose

Next, we assessed whether the C1184 preparation has to be supplemented with β-glucosidase, β-xylosidase or β-mannosidase activities to convert the major polysaccharides forming the holocellulose content of lignocellulose to sugar monomers. For this, we analysed the product profile of the C1184 preparation when cleaving linear, unsubstituted oligosaccharides, i.e., COS, XOS, MOS and LOS. TLC results showed that COS with DP 2–4 were converted mainly to glucose, with some cellobiose remaining in the reaction hydrolysate (Fig. 4A). This indicates that C1184 does possess some β-glucosidase activity, but it may not be satisfactory at industrially relevant substrate concentrations (i.e., above the 1 mg/mL tested here). Although *Aspergillus* sp., especially *A. niger*, are among the best fungal sources for β-glucosidase production (Sternberg et al. 1977), our data, in agreement with the manufacturer’s information, indicate that the C1184 preparation has low β-glucosidase activity and requires supplementation with an external β-glucosidase source.

On the other hand, XOS with DP 2–5 were also hydrolysed by the C1184 preparation to xylose, with small amounts of xylotriose remaining in the reactions with longer XOS, especially with xylotriose and xylopentaose (Fig. 4B). As pure xylanases (e.g. GH11 xylanases) produce mostly xylobiose (i.e., not xylose) from XOS with DP 3–6 (Aiewviriyasakul et al. 2021; Zhang et al. 2021), this is a clear indication that the C1184 preparation contains enzymes with β-xylosidase activity. Our data are not surprising as both β-xylosidases and β-glucosidases occur commonly in fungal secretomes, such as those of *Aspergillus* sp. (Borin et al. 2015; de Vries and Visser 2001). Given that xylosidase converted xylobiose completely to xylose, we assumed that it would not be necessary to supplement the C1184 preparation with β-xylosidase during xylan saccharification studies.

Furthermore, the hydrolysis of longer MOS (with DP 4–5) to mannotriose, mannobiose and some amounts of mannose (Fig. 4A) confirms the presence of endo-β-(1 → 4)-mannanases in the C1184 preparation and indicates that the galactosyl substitutions in carob galactomannan, indeed, sterically hindered depolymerisation of the β-(1 → 4)-mannan backbone by these β-mannanases (shown in Fig. 2D). However, the fact that shorter MOS (mannobiose and mannotriose) were not hydrolysed effectively to mannose monomers suggests a limited amount of β-mannosidases present in the C1184 preparation (Fig. 4A).

As the initial activity tests revealed that the C1184 preparation can cleave algal β-(1 → 3)-glucan, we set up similar reactions with LOS to confirm the presence of β-(1 → 3)-glucanase activity and to assess if the C1184 preparation possesses β-(1 → 3)-glucosidase activity to convert the released LOS to glucose. Indeed, the β-(1 → 3)-glucanases present in the C1184 preparation hydrolysed the longer oligosaccharides, with DP 3–5. However, laminaribiose was left nearly intact (Fig. 4C), indicating limited, if any, β-(1 → 3)-glucosidase activity in the enzyme preparation.

### Characterisation of mango seed husk (MSH)

**Fig. 5:**
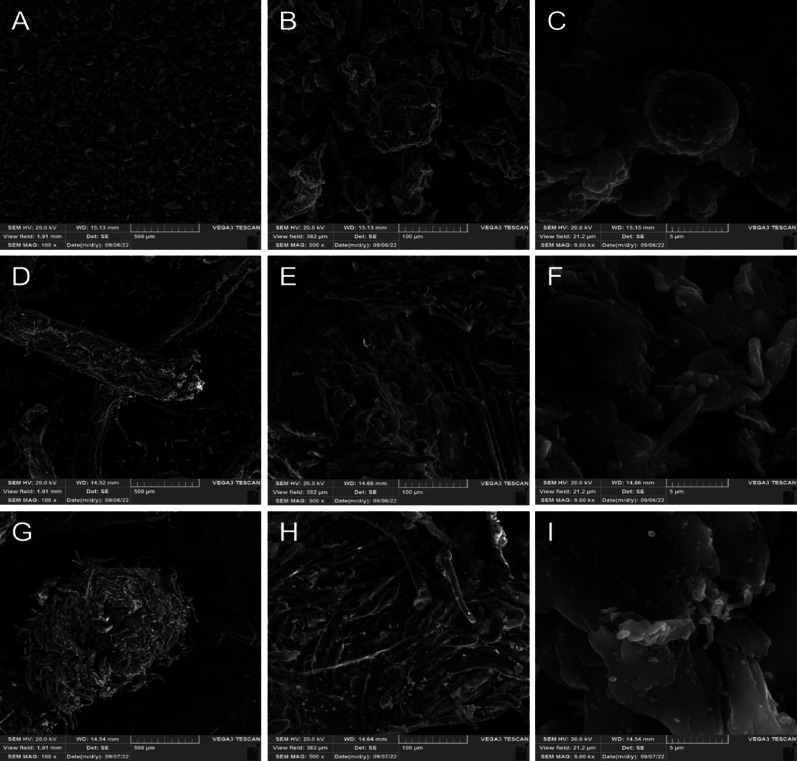
Topological analysis of Avicel and the fine and coarse fractions of ground MSH using SEM. Images of Avicel (**A**–**C**) and the fine (**D**–**F**) and coarse (**G**–**I**) fractions of ground MSH were recorded with 100× (**A**, **D**, **G**), 500× (**B**, **E**, **H**) and 9000× (**C**, **F**, **I**) magnifications with scale bars of 500 µm, 100 µm and 5 µm, respectively

The two ground MSH fractions, with fine powder and coarse particles, were subjected to topological analysis using SEM, with Avicel as a control. The canonical topological structure of Avicel at 100× and 500× magnification (Fig. 5A–B) displays distinct, compact aggregates of short fibres with a relatively homogeneous particle size (50–100 µm in diameter). At 9000× magnification of the surface area of these fibre aggregates (Fig. 5C), we observed sphere-like biomass particles with about 5 µm in diameter that have round formations with about 1 µm in diameter. Such round formations are seldom observed or reported on the surface of Avicel. Avicel is a pure cellulose with high crystallinity, void of lignin or other compounds that may form precipitate on the surface (Gomide et al. 2019; Sannigrahi et al. 2011). A potential reason for these round formations could be aggregation/deposition of shorter cellulose fibrils upon removal of lignin or hemicellulose from the plant cell wall (Cosgrove 2022; Gomide et al. 2019). Its aggregated nature makes Avicel more resistant toward enzymatic hydrolysis primarily due to reduced average pore size, limiting the penetration of enzymes into the fibres (Cosgrove 2022; Peciulyte et al. 2015; Van Dyk and Pletschke 2012).

Regarding MSH, studies have suggested that cellulose in MSH differs from that of Avicel (β-cellulose, with shorter chains) because it consists of α-cellulose (longer cellulose chains and higher DP) (Henrique et al. 2013). Indeed, while both Avicel and MSH samples contained clear fibre-like structures (visible at 100× and 500× magnifications in Fig. 5), the MSH samples had more elongated fibre-like structures with a rough surface. Contrary to Avicel, at 9000× magnification, the biomass surface in the MSH fractions displayed a smooth, scales-like structure with minimal round, deposit-like formations. Furthermore, the fine and coarse fractions of MSH had distinct topological properties, which resulted from variations in the structure of the fibre networks. The coarse MSH samples contained spherical agglomerates of fibres, with a net-like pattern of fibres at an angled orientation and fibre-like structures sticking outward (Fig. 5G, H). On the other hand, the fine MSH samples contained longer, linear fibres with parallel fibre orientation (Fig. 5D, E). At 9000× , the surface of the coarse MSH fraction carried a lot of small round particles attached to the surface, with a few fibre-like structures sticking out (Fig. 5H). Note that the fine fraction of MSH also contained such small round particles, but in much smaller numbers (Fig. 5F). These particles could potentially be due to the mineral content of the fibres (see Table 2 and an explanation further below, as well as Figure S4). Phloroglucinol staining confirmed that the (surface of the) coarse fraction had considerably higher lignin content than the (surface of the) fine fraction of MSH (Figure S3).

**Table 2:** Elemental analysis of Avicel and ground MSH fractions using energy dispersive X-ray spectrometry (EDS)

Elements	Avicel (w/w%)*	Ground MSH, fine fraction (w/w%)*	Ground MSH, coarse fraction (w/w%)*
O	97.8 ± 0.6	79.4 ± 0.6	72.8 ± 0.4
K	b.d.l.	6.4 ± 0.3	12.7 ± 0.2
Ca	b.d.l.	4.8 ± 0.3	11.5 ± 0.4
Mg	b.d.l.	4.2 ± 0.3	1.1 ± 0.1
Cu	2.2 ± 0.6	b.d.l.	b.d.l.
S	b.d.l.	1.9 ± 0.2	1.0 ± 0.2
Cl	b.d.l.	1.6 ± 0.2	0.6 ± 0.1
P	b.d.l.	1.0 ± 0.2	b.d.l.
Si	b.d.l.	0.7 ± 0.2	0.3 ± 0.1

*Values represent the mean percentage atomic weight of the detected element ± standard deviation (σ). Carbon was excluded from the analysis because it was used to coat the sample to make it conductive

b.d.l.: below detection limit

**Fig. 6:**
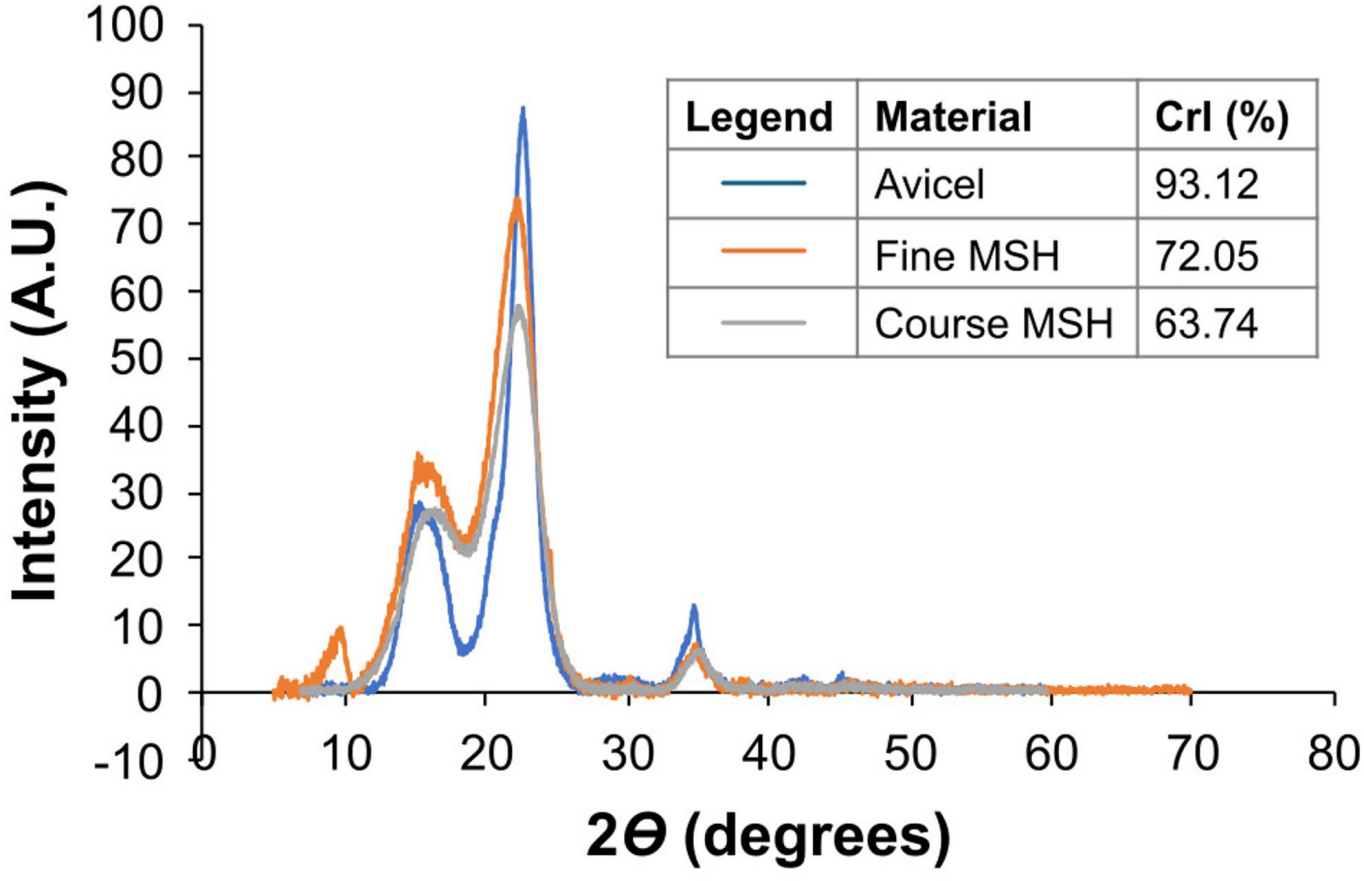
XRD diffractogram of Avicel and the fine and coarse fractions of ground MSH. The crystallinity indices (CrI) for Avicel (blue line) and the fine (orange line) and coarse (grey line) fractions of ground MSH are indicated in the legend

In addition to the distinct morphological differences, the fine and coarse fractions of ground MSH differed in their elemental surface compositions, as shown by EDS (Table 2). As expected, carbon (C) and oxygen (O) constituted the highest percentage of elements detected in the Avicel and MSH samples. In particular, the coarse fraction of MSH contained much higher amounts of K and Ca, while the fine fraction of MSH was richer in Mg, S, Cl, P and Si (Table 2). Additionally, XRD analysis of the crystallinity index (CrI) showed that, among the three biomass samples, Avicel (also known as microcrystalline cellulose) had the highest crystallinity, about 93% CrI. Figure 6 shows that the fine fraction of MSH had higher crystallinity (72% CrI) than the coarse fraction of MSH (64% CrI), which is consistent with the lower lignin content in the fine MSH fraction (Figure S3).

### Production of glucose and phenolics from MSH with the C1184 preparation supplemented with feruloyl and glucuronoyl esterases

Feruloyl and glucuronoyl esterases improve the hydrolysis of the lignocellulosic biomass by removing ester bonds that crosslink lignin and hemicellulose, xylan in particular (d'Errico et al. 2016; Dilokpimol et al. 2016). To determine the effects of cleaving such ester bonds on the release of glucose or phenolic compounds from MSH, the untreated fine and coarse fractions of ground MSH were subjected to enzyme treatment using the C1184 preparation alone or supplemented with esterase enzymes (*Ct*FAE, *rm*FAE or *Rf*GE) on a 1:1 (w/w) enzyme ratio (Table 3). Interestingly, there was little difference in the glucose release by the C1184 preparation when acting alone on the two MSH fractions despite the lower lignin content in the fine fraction (Figure S3). Notably, the C1184 preparation seemed to lack feruloyl and glucuronoyl esterase activities, as indicated by the absence of phenolics released without feruloyl and glucuronoyl esterase supplementation. On the other hand, the tested esterases were active on MSH and were able to release phenolic compounds from the MSH fractions. Interestingly, all three esterases released about three to five times more phenolics from the fine fraction of MSH with lower levels of lignin, compared to the coarse fraction with higher lignin content. This could suggest that lignin that forms lignin–carbohydrate complexes may be less condensed in the fine fraction than in the coarse fraction and could therefore be more readily released.

**Table 3:** Glucose and phenolics released from ground MSH using the C1184 preparation supplemented with esterases

Substrate	Enzymes	Glucose % yield (w/w of total biomass)^a,b^	Total phenolics release (µg/mL)^a^
Ground MSH, fine fraction	C1184	10.3 ± 0.2	b.d.l.^**c**^
	C1184 + *Ct*FAE	8.9 ± 1.1*	156 ± 15*
	C1184 + *rm*FAE	8.4 ± 1.5*	139 ± 12*
	C1184 + *Rf*GE	19.5 ± 0.6*	171 ± 10*
Ground MSH, course fraction	C1184	10.3 ± 0.1	b.d.l.^c^
	C1184 + *Ct*FAE	9.2 ± 0.1*	56 ± 19*
	C1184 + *rm*FAE	9.4 ± 0.1*	38 ± 4*
	C1184 + *Rf*GE	16.4 ± 2.3*	36 ± 5*

^a^Experiments were performed in triplicate, and values represent means ± standard deviation

^b^Glucose yields are expressed as anhydro form, and thus glucose yields (%) are presented as w/w of the total glucan in the biomass

^c^b.d.l.: below detection limit

*Indicate the significant difference in glucose and phenolics concentration released by applying C1184 cellulase preparation or the combination of C1184 and esterase enzymes to MSH biomass

Lignin is linked to hemicellulose content through feruloyl and glucuronoyl ester bonds (Mnich et al. 2020). Several studies have shown that cleaving such ester bonds facilitates the depolymerisation of xylan (Malgas et al. 2019), which can indirectly facilitate the depolymerisation of cellulose (d'Errico et al. 2016; Larsbrink and Lo Leggio 2023) or potentially even other polysaccharides that are intertwined with cellulose and xylan, such as glucomannan (Várnai et al. 2011). In our case, replacing 50% of the C1184 preparation with one of the feruloyl esterases (*Ct*FAE or *rm*FAE) reduced the sugar release (obviously, as the cellulase loading was halved), while it led to a release of phenolic compounds into solution (Table 3). Remarkably, replacing 50% of the C1184 preparation with the glucuronoyl esterase (*Rf*GE) nearly doubled the glucose release on both the fine and coarse fractions of MSH, despite having half the amount of cellulases in the reaction. This finding indicates that lignin–carbohydrate linkages formed via glucuronoyl esters may be much more prevalent than hydroxycinnamoyl esters in MSH, and that their cleavage is much more important for the total saccharification of MSH than the cleavage of hydroxycinnamoyl esters. Regarding hydroxycinnamoyl esters, their hydrolysis by *Ct*FAE and *rm*FAE had a higher positive impact on the phenolic compounds release from the coarse fraction, even though about three times less phenolic compounds were solubilised from the coarse fraction of MSH compared to the fine fraction. This finding further supports the notion that lignin in the coarse fraction of MSH may be more condensed, limiting the release of phenolics into solution after cleavage of hydroxycinnamoyl esters.

Glucuronoyl esterase facilitates the depolymerisation of cellulose and xylan in synergy with cellulases and xylanases; however, the interplay between glucuronoyl esterases, xylanases and cellulases needs further elucidation. Arnling Bååth et al. (2018) proposed that glucuronoyl esterases could decouple the (methylated or non-methylated) glucuronoyl group from lignin, exposing more cleavage sites for xylanases, which create subsequent cleaving sites for cellulases. It is noteworthy that Arnling Bååth et al. (2019) have observed inhibition of *Tt*CE15A from *Teredinibacter turnerae* by ferulic, *p*-coumaric and salicylic acid, confirming that glucuronoyl esterase interacts with both the phenolic compounds of lignin and carbohydrate components of the plant cell wall. The inhibitor concentrations required to reduce the reaction rate by 50% (IC_50_ values) for ferulic, *p*-coumaric and salicylic acid corresponded to 107, 77 and 136 µg/mL, respectively (Arnling Bååth et al. 2019). Considering that *Rf*GE released similar overall amounts of phenolic compounds from MSH, it would be interesting to study the types of phenolic compounds released and their impact on the action of *Rf*GE in a follow-up study.

### Saccharification of alkali-pretreated MSH with the C1184 preparation

**Fig. 7:**
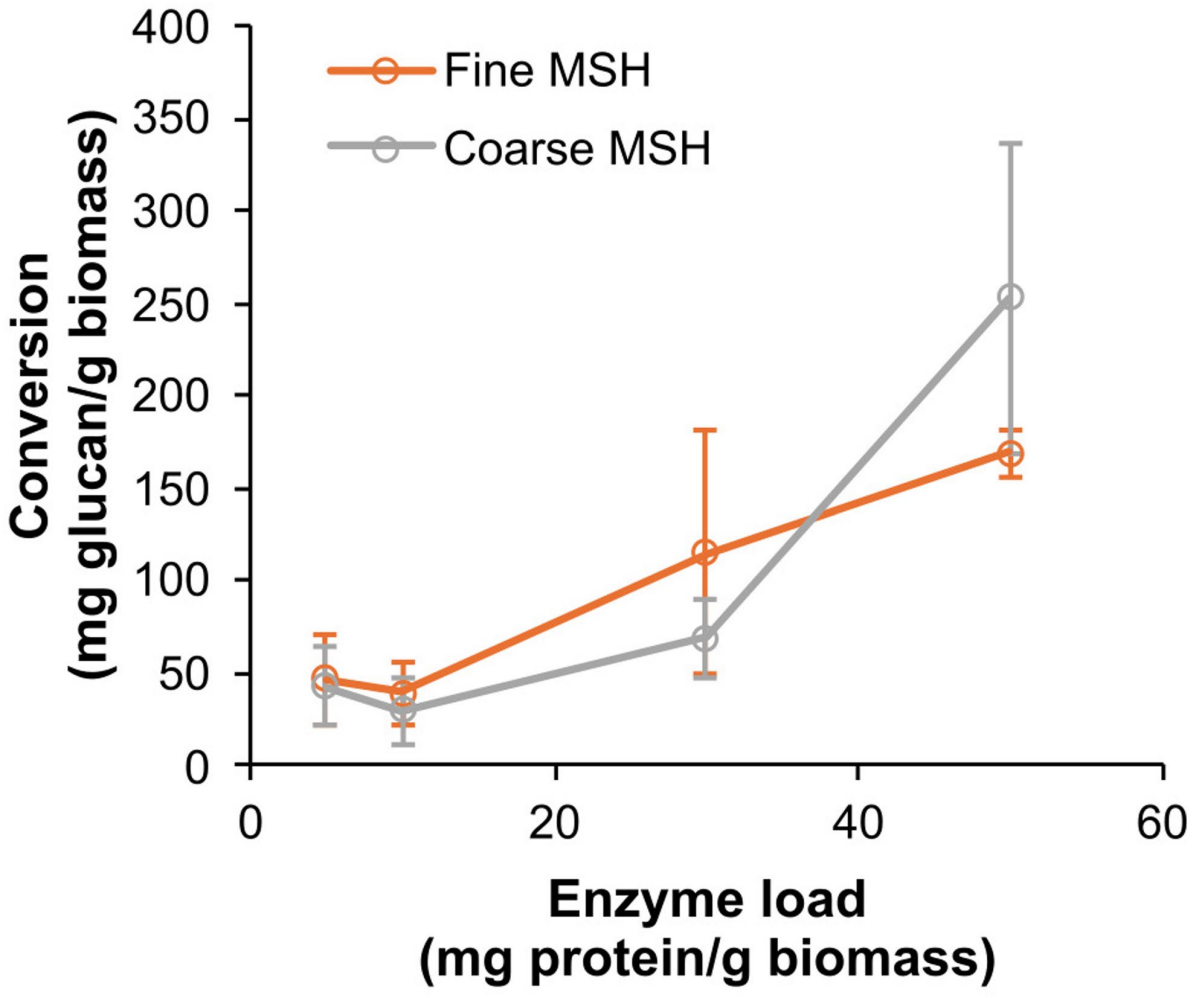
Saccharification of the alkali-pretreated fractions of MSH with the C1184 preparation supplemented with β-glucosidase. Reactions were set up with 1% (w/v) alkali-pretreated fine (orange circles) or coarse (gray circles) fraction of MSH and 5–50 mg protein/g biomass cellulase mixture containing 90% (w/w) C1184 preparation and 10% (w/w) β-glucosidase and incubated at 40 °C for 24 h. The experiments were performed in triplicates; the values represent means, and the error bars standard deviation

The fine and coarse fractions of ground MSH were subjected to further saccharification studies with the C1184 preparation after pretreatment with 20% NaOH, using the following enzyme loadings: 5, 10, 30 and 50 mg/mg dry biomass. The C1184 preparation was supplemented with 10% β-glucosidase to ensure conversion of the COS to glucose due to the limited β-glucosidase activity observed in the C1184 preparation (see section "Oligosaccharides Hydrolysis patterns produced by the C1184 preparation"). Feruloyl and glucuronoyl esterases were not tested in this setup because the alkali pretreatment saponifies (and thus removes) the ester bonds targeted by these enzymes (Ralph et al. 1994). Figure 7 shows that the C1184 preparation solubilised similar amounts of cellulose at the lowest enzyme doses (5 and 10 mg/g biomass), with slightly higher glucose released from the alkali-pretreated fine fraction of MSH compared to the alkali-pretreated coarse fraction. There was some difference in the efficiency of the C1184 preparation toward the two alkali-treated MSH fractions at 30 and 50 mg/g biomass enzyme loadings; however, due to high variation, the differences were not significant. While the C1184 preparation supplemented with 10% (w/w) β-glucosidase was able to produce glucose from the alkali-pretreated MSH samples, the yields after 24 h remained rather low [< 30% (w/w) of the complete biomass, including lignin and hemicelluloses] despite the high enzyme loadings (50 mg protein/g biomass). One reason for the low efficiency could be the inferior cellulolytic and xylanolytic activity of the preparation as compared to other commercial cellulase cocktails (see our discussion at the end of section "Biochemical characteristics and enzyme kinetics of cellulase, xylanase and mannanase activities in the C1184 preparation""). Thus, considerable process optimisation is needed before the C1184 preparation can be used for glucose production from MSH.

## Concluding remarks

Our findings underscore the importance of understanding enzyme compositions in enzyme cocktails or preparations to identify suitable applications and potential products. Even though the C1184 preparation is marketed as a cellulase by the manufacturer, we showed that xylanolytic enzymes were highly expressed and form the most active component of the enzyme preparation. Given that the C1184 preparation lacks glucuronoyl and feruloyl esterases, it could potentially be used to produce lignin–carbohydrate complexes, e.g. for the determination of lignin–xylan cross-links in MSH, or decorated xylo-oligosaccharides with bioactive and antioxidant properties.

This study corroborates that MSH, a by-product of the mango industry, is a promising source of value-added products, including glucose and phenolics. Our data show that differences in the physical appearance and chemical composition of MSH fractions influence biomass valorization potential. The fine fraction of MSH, with parallel cellulose fibres, lower lignin content and presumably lower extent of lignin condensation, yielded higher phenolic compound extraction compared to the coarse fraction. Thus, separating MSH fractions based on particle size prior to processing could improve not only product recovery, for e.g. phenolic compounds, but potentially also uniformity for fibres and nanocellulose for biomaterial applications.

Regarding plant cell wall structure, our data indicate that lignin–carbohydrate linkages via both feruloyl and glucuronoyl esters contribute to recalcitrance in MSH. However, the glucuronoyl esterase had a remarkably bigger impact on glucose release from ground MSH by the C1184 preparation than the feruloyl esterases, suggesting that targeting glucuronoyl esters can alleviate biomass recalcitrance in MSH more effectively than targeting hydroxycinnamoyl esters. The substantial impact of glucuronoyl esterase substitution on cellulose saccharification highlights how the secretion of a single enzyme component by microorganisms can alleviate the metabolic burden and reduce overall protein needs in both natural and biotechnological processes.

## Supplementary Information

Below is the link to the electronic supplementary material.

Supplementary Material 1

## Data Availability

All data generated or analysed during this study are included in this published article and its supplementary information files.

## Abbreviations

AA: Auxiliary activities
CAZyme: Carbohydrate-active enzyme
CE: Carbohydrate esterase
CMC: Carboxymethylcellulose
COS: Cello-oligosaccharides
CrI: Crystallinity index
DNS: 3,5-Dinitrosalicylic acid
DP: Degree of polymerisation
EDS: Energy-dispersive X-ray spectroscopy
FAE: Feruloyl esterase
GE: Glucuronoyl esterase
GH: Glycoside hydrolase
KGM: Konjac glucomannan
LOS: Laminari-oligosaccharides
LPMO: Lytic polysaccharide monooxygenase
MLG: Mixed-linkage β-d-(1 → 3),(1 → 4)-glucan
MOS: Manno-oligosaccharides
MSH: Mango seed husk
SDS-PAGE: Sodium dodecyl sulfate–polyacrylamide gel electrophoresis
SEM: Scanning electron microscopy
TLC: Thin-layer chromatography
WAX: Wheat arabinoxylan
XOS: Xylo-oligosaccharides
XRD: X-ray diffraction

## References

[CR1] Ademark P, Varga A, Medve J, Harjunpää V, Torbjörn D, Tjerneld F, Stålbrand H (1998) Softwood hemicellulose-degrading enzymes from *Aspergillus niger*: purification and properties of a β-mannanase. J Biotechnol 63(3):199–210. 10.1016/S0168-1656(98)00086-8 9803534 10.1016/s0168-1656(98)00086-8

[CR2] Aguilar-Pontes MV, Brandl J, McDonnell E, Strasser K, Nguyen TTM, Riley R, Mondo S, Salamov A, Nybo JL, Vesth TC, Grigoriev IV (2018) The gold-standard genome of *Aspergillus niger* NRRL 3 enables a detailed view of the diversity of sugar catabolism in fungi. Stud Mycol 91:61–78. 10.1016/j.simyco.2018.10.001 30425417 10.1016/j.simyco.2018.10.001 PMC6231085

[CR3] Aiewviriyasakul K, Bunterngsook B, Lekakarn H, Sritusnee W, Kanokratana P, Champreda V (2021) Biochemical characterization of xylanase GH11 isolated from *Aspergillus niger* BCC14405 (XylB) and its application in xylooligosaccharide production. Biotechnol Lett 43(12):2299–2310. 10.1007/s10529-021-03202-1 34718907 10.1007/s10529-021-03202-1

[CR4] Ainsworth EA, Gillespie KM (2007) Estimation of total phenolic content and other oxidation substrates in plant tissues using Folin–Ciocalteu reagent. Nat Protoc 2(4):875–877. 10.1038/nprot.2007.102 17446889 10.1038/nprot.2007.102

[CR5] Amir A, Arif M, Pande V (2013) Purification and characterization of xylanase from *Aspergillus fumigatus* isolated from soil. Afr J Biotechnol 12(20):3049–3057. 10.5897/AJB2013.12152

[CR6] Andrade LA, Barrozo MAS, Vieira LGM (2016) Thermo-chemical behavior and product formation during pyrolysis of mango seed shell. Ind Crops Prod 85:174–180. 10.1016/j.indcrop.2016.03.004

[CR7] Arnaud MB, Cerqueira GC, Inglis DO, Skrzypek MS, Binkley J, Chibucos MC, Crabtree J, Howarth C, Orvis J, Shah P, Wymore F (2012) The *Aspergillus* Genome Database (AspGD): recent developments in comprehensive multispecies curation, comparative genomics and community resources. Nucleic Acids Res 40(D1):D653–D659. 10.1093/nar/gkr875 22080559 10.1093/nar/gkr875 PMC3245136

[CR8] Arnling Bååth J, Mazurkewich S, Knudsen RM, Poulsen J-CN, Olsson L, Lo Leggio L, Larsbrink J (2018) Biochemical and structural features of diverse bacterial glucuronoyl esterases facilitating recalcitrant biomass conversion. Biotechnol Biofuels 11(1):213. 10.1186/s13068-018-1213-x 30083226 10.1186/s13068-018-1213-x PMC6069808

[CR9] Arnling Bååth J, Mazurkewich S, Poulsen J-CN, Olsson L, Lo Leggio L, Larsbrink J (2019) Structure–function analyses reveal that a glucuronoyl esterase from *Teredinibacter turnerae* interacts with carbohydrates and aromatic compounds. J Biol Chem 294(16):6635–6644. 10.1074/jbc.RA119.007831 30814248 10.1074/jbc.RA119.007831 PMC6484129

[CR10] Balan V, Chiaramonti D, Kumar S (2013) Review of US and EU initiatives toward development, demonstration, and commercialization of lignocellulosic biofuels. Biofuels Bioprod Bioref 7(6):732–759. 10.1002/bbb.1436

[CR11] Bello F, Chimphango A (2021) Optimization of lignin extraction from alkaline treated mango seed husk by high shear homogenization-assisted organosolv process using response surface methodology. Int J Biol Macromol 167:1379–1392. 10.1016/j.ijbiomac.2020.11.092 33202271 10.1016/j.ijbiomac.2020.11.092

[CR12] Bello F, Chimphango A (2022) Tailor-made conversion of mango seed husks to obtain hemicellulose suitable for the production of thermally stable films. Waste Biomass Valorization 13(1):719–737. 10.1007/s12649-021-01506-x

[CR13] Berka RM, Grigoriev IV, Otillar R, Salamov A, Grimwood J, Reid I, Ishmael N, John T, Darmond C, Moisan MC, Henrissat B (2011) Comparative genomic analysis of the thermophilic biomass-degrading fungi *Myceliophthora thermophila* and *Thielavia terrestris*. Nat Biotechnol 29(10):922–927. 10.1038/nbt.1976 21964414 10.1038/nbt.1976

[CR14] Bhushan B, Pal A, Kumar S, Jain V (2015) Biochemical characterization and kinetic comparison of encapsulated haze removing acidophilic xylanase with partially purified free xylanase isolated from *Aspergillus flavus* MTCC 9390. J Food Sci Technol 52(1):191–200. 10.1007/s13197-013-1013-z

[CR15] Bischof RH, Ramoni J, Seiboth B (2016) Cellulases and beyond: the first 70 years of the enzyme producer *Trichoderma reesei*. Microb Cell Fact 15(1):106. 10.1186/s12934-016-0507-6 27287427 10.1186/s12934-016-0507-6 PMC4902900

[CR16] Borin GP, Sanchez CC, de Souza AP, de Santana ES, de Souza AT, Paes Leme AF, Squina FM, Buckeridge M, Goldman GH, Oliveira JV (2015) Comparative secretome analysis of *Trichoderma reesei* and *Aspergillus niger* during growth on sugarcane biomass. PLoS ONE 10(6):e0129275. 10.1371/journal.pone.0129275 26053961 10.1371/journal.pone.0129275 PMC4460134

[CR17] Bradford MM (1976) A rapid and sensitive method for the quantitation of microgram quantities of protein utilizing the principle of protein-dye binding. Anal Biochem 72:248–254. 10.1006/abio.1976.9999 942051 10.1016/0003-2697(76)90527-3

[CR18] Chandel AK, Garlapati VK, Singh AK, Antunes FAF, da Silva SS (2018) The path forward for lignocellulose biorefineries: bottlenecks, solutions, and perspective on commercialization. Bioresour Technol 264:370–381. 10.1016/j.biortech.2018.06.004 29960825 10.1016/j.biortech.2018.06.004

[CR19] Chen Y, Luo H, Gao A, Zhu M (2012) Extraction of polysaccharides from mango (*Mangifera indica* Linn.) seed by response surface methodology and identification of their structural characteristics. Food Anal Methods 5(4):800–806. 10.1007/s12161-011-9312-3

[CR20] Chinedu SN, Nwinyi OC, Okafor UA, Okochi VI (2011) Kinetic study and characterization of 1,4-β-endoglucanase of *Aspergillus niger* ANL301. Dyn Biochem Process Biotechnol Mol Biol 5(2):41–46

[CR21] Choudhary P, Devi TB, Tushir S, Kasana RC, Popatrao DS, K N (2023) Mango seed kernel: a bountiful source of nutritional and bioactive compounds. Food Bioprocess Technol 16(2):289–312. 10.1007/s11947-022-02889-y

[CR22] Chylenski P, Forsberg Z, Ståhlberg J, Várnai A, Lersch M, Bengtsson O, Saebo S, Horn SJ, Eijsink VGH (2017) Development of minimal enzyme cocktails for hydrolysis of sulfite-pulped lignocellulosic biomass. J Biotechnol 246:16–23. 10.1016/j.jbiotec.2017.02.009 28219736 10.1016/j.jbiotec.2017.02.009

[CR23] Cosgrove DJ (2022) Building an extensible cell wall. Plant Physiol 189(3):1246–1277. 10.1093/plphys/kiac184 35460252 10.1093/plphys/kiac184 PMC9237729

[CR24] de Vries RP (2003) Regulation of *Aspergillus* genes encoding plant cell wall polysaccharide-degrading enzymes; relevance for industrial production. Appl Microbiol Biotechnol 61(1):10–20. 10.1007/s00253-002-1171-9 12658510 10.1007/s00253-002-1171-9

[CR25] de Vries RP, Visser J (2001) *Aspergillus* enzymes involved in degradation of plant cell wall polysaccharides. Microbiol Mol Biol Rev 65(4):497–522. 10.1128/mmbr.65.4.497-522.2001 11729262 10.1128/MMBR.65.4.497-522.2001 PMC99039

[CR26] Debnath B, Haldar D, Purkait MK (2021) Potential and sustainable utilization of tea waste: a review on present status and future trends. J Environ Chem Eng 9(5):106179. 10.1016/j.jece.2021.106179

[CR27] d’Errico C, Börjesson J, Ding H, Krogh KB, Spodsberg N, Madsen R, Monrad RN (2016) Improved biomass degradation using fungal glucuronoyl-esterases-hydrolysis of natural corn fiber substrate. J Biotechnol 219:117–123. 10.1016/j.jbiotec.2015.12.024 26712478 10.1016/j.jbiotec.2015.12.024

[CR28] Dilokpimol A, Mäkelä MR, Aguilar-Pontes MV, Benoit-Gelber I, Hildén KS, de Vries RP (2016) Diversity of fungal feruloyl esterases: updated phylogenetic classification, properties, and industrial applications. Biotechnol Biofuels 9(1):231. 10.1186/s13068-016-0651-6 27795736 10.1186/s13068-016-0651-6 PMC5084320

[CR29] Ding C, Li M, Hu Y (2018) High-activity production of xylanase by *Pichia stipitis*: purification, characterization, kinetic evaluation and xylooligosaccharides production. Int J Biol Macromol 117:72–77. 10.1016/j.ijbiomac.2018.05.128 29792957 10.1016/j.ijbiomac.2018.05.128

[CR30] Dobrev GT, Zhekova BY (2012) Biosynthesis, purification and characterization of endoglucanase from a xylanase producing strain *Aspergillus niger* B03. Braz J Microbiol 43(1):70–77. 10.1590/s1517-83822012000100008 24031805 10.1590/S1517-83822012000100008 PMC3768974

[CR31] Drula E, Garron M-L, Dogan S, Lombard V, Henrissat B, Terrapon N (2022) The carbohydrate-active enzyme database: functions and literature. Nucleic Acids Res 50(D1):D571–D577. 10.1093/nar/gkab1045 34850161 10.1093/nar/gkab1045 PMC8728194

[CR32] Elizalde-González MP, Hernández-Montoya V (2007) Characterization of mango pit as raw material in the preparation of activated carbon for wastewater treatment. Biochem Eng J 36(3):230–238. 10.1016/j.bej.2007.02.025

[CR33] Frederick MM, Kiang C-H, Frederick JR, Reilly PJ (1985) Purification and characterization of endo-xylanases from *Aspergillus niger.* I. Two isozymes active on xylan backbones near branch points. Biotechnol Bioeng 27(4):525–532. 10.1002/bit.260270420 18553703 10.1002/bit.260270420

[CR34] Gomide FTF, da Silva ASA, da Silva Bon EP, Alves TLM (2019) Modification of microcrystalline cellulose structural properties by ball-milling and ionic liquid treatments and their correlation to enzymatic hydrolysis rate and yield. Cellulose 26(12):7323–7335. 10.1007/s10570-019-02578-8

[CR35] Henrique MA, Silvério HA, Flauzino Neto WP, Pasquini D (2013) Valorization of an agro-industrial waste, mango seed, by the extraction and characterization of its cellulose nanocrystals. J Environ Manage 121:202–209. 10.1016/j.jenvman.2013.02.054 23542530 10.1016/j.jenvman.2013.02.054

[CR36] Hurst PL, Nielsen J, Sullivan PA, Shepherd MG (1977) Purification and properties of a cellulase from *Aspergillus niger*. Biochem J 165(1):33–41. 10.1042/bj1650033 19015 10.1042/bj1650033 PMC1164865

[CR37] Janusz G, Pawlik A, Sulej J, Świderska-Burek U, Jarosz-Wilkołazka A, Paszczyński A (2017) Lignin degradation: microorganisms, enzymes involved, genomes analysis and evolution. FEMS Microbiol Rev 41(6):941–962. 10.1093/femsre/fux049 29088355 10.1093/femsre/fux049 PMC5812493

[CR38] Kabel MA, van der Maarel MJ, Klip G, Voragen AG, Schols HA (2006) Standard assays do not predict the efficiency of commercial cellulase preparations towards plant materials. Biotechnol Bioeng 93(1):56–63. 10.1002/bit.20685 16196058 10.1002/bit.20685

[CR39] Kittiphoom S (2012) Utilization of mango seed. Int Food Res J 19:1325–1335

[CR40] Laemmli UK (1970) Cleavage of structural proteins during the assembly of the head of bacteriophage T4. Nature 227(5259):680–685. 10.1038/227680a0 5432063 10.1038/227680a0

[CR41] Larsbrink J, Lo Leggio L (2023) Glucuronoyl esterases – enzymes to decouple lignin and carbohydrates and enable better utilization of renewable plant biomass. Essays Biochem 67(3):493–503. 10.1042/ebc20220155 36651189 10.1042/EBC20220155 PMC10154605

[CR42] Mafa MS, Dirr HW, Malgas S, Krause RWM, Rashamuse K, Pletschke BI (2020a) A novel dimeric exoglucanase (GH5_38): biochemical and structural characterisation towards its application in alkyl cellobioside synthesis. Molecules 25(3):746. 10.3390/molecules25030746 32050450 10.3390/molecules25030746 PMC7036808

[CR43] Mafa MS, Malgas S (2023) Towards an understanding of the enzymatic degradation of complex plant mannan structures. World J Microbiol Biotechnol 39(11):302. 10.1007/s11274-023-03753-7 37688610 10.1007/s11274-023-03753-7 PMC10492685

[CR44] Mafa MS, Malgas S, Bhattacharya A, Rashamuse K, Pletschke BI (2020b) The effects of alkaline pretreatment on agricultural biomasses (corn cob and sweet sorghum bagasse) and their hydrolysis by a termite-derived enzyme cocktail. Agronomy 10(8):1211. 10.3390/agronomy10081211

[CR45] Mafa MS, Pletschke BI, Malgas S (2021) Defining the frontiers of synergism between cellulolytic enzymes for improved hydrolysis of lignocellulosic feedstocks. Catalysts 11(11):1343. 10.3390/catal11111343

[CR46] Malgas S, Mafa MS, Mkabayi L, Pletschke BI (2019) A mini review of xylanolytic enzymes with regards to their synergistic interactions during hetero-xylan degradation. World J Microbiol Biotechnol 35(12):187. 10.1007/s11274-019-2765-z 31728656 10.1007/s11274-019-2765-z

[CR47] Manhongo TT, Chimphango A, Thornley P, Röder M (2021) Techno-economic and environmental evaluation of integrated mango waste biorefineries. J Clean Prod 325:129335. 10.1016/j.jclepro.2021.129335

[CR48] Martinez D, Berka RM, Henrissat B, Saloheimo M, Arvas M, Baker SE, Chapman J, Chertkov O, Coutinho PM, Cullen D, Danchin EG (2008) Genome sequencing and analysis of the biomass-degrading fungus *Trichoderma reesei* (syn. *Hypocrea jecorina*). Nat Biotechnol 26(5):553–560. 10.1038/nbt1403 18454138 10.1038/nbt1403

[CR49] McCleary BV, Matheson NK (1983) Action patterns and substrate-binding requirements of β-D-mannanase with mannosaccharides and mannan-type polysaccharides. Carbohydr Res 119:191–219. 10.1016/0008-6215(83)84056-7

[CR50] Miller GL (1959) Use of dinitrosalicylic acid reagent for determination of reducing sugar. Anal Chem 31(3):426–428. 10.1021/ac60147a030

[CR51] Mnich E, Bjarnholt N, Eudes A, Harholt J, Holland C, Jørgensen B, Larsen FH, Liu M, Manat R, Meyer AS, Mikkelsen JD (2020) Phenolic cross-links: building and de-constructing the plant cell wall. Nat Prod Rep 37(7):919–961. 10.1039/c9np00028c 31971193 10.1039/c9np00028c

[CR52] Mohotloane MM, Alexander O, Adoons VN, Pletschke BI, Mafa MS (2024) Peroxidase application reduces microcrystalline cellulose recalcitrance towards cellulase hydrolysis in model cellulose substrates and rooibos biomass. Carbohydr Polym Technol Appl 7:100426. 10.1016/j.carpta.2024.100426

[CR53] Mohotloane MM, Alexander O, Pletschke BI, Mafa MS (2023) Horseradish peroxidase delignification of fermented rooibos modifies biomass structural and chemical properties and improves holocellulolytic enzyme cocktail efficacy. Biologia 78(7):1943–1959. 10.1007/s11756-023-01424-4

[CR54] Monclaro AV, Aquino EN, Faria RF, Filho EXF, Ricart CAO, Freitas SM, Midorikawa GEO, Miller RNG, Michelin M, Polizeli MLTM (2016) Characterization of multiple xylanase forms from *Aspergillus tamarii* resistant to phenolic compounds. Mycosphere 7(10):1554–1567. 10.5943/mycosphere/si/3b/7

[CR55] Monclaro AV, Recalde GL, da Silva FG, de Freitas SM, Ferreira Filho EX (2019) Xylanase from *Aspergillus tamarii* shows different kinetic parameters and substrate specificity in the presence of ferulic acid. Enzyme Microb Technol 120:16–22. 10.1016/j.enzmictec.2018.09.009 30396395 10.1016/j.enzmictec.2018.09.009

[CR56] Mujtaba M, Fernandes Fraceto L, Fazeli M, Mukherjee S, Savassa SM, de Araujo Meiros G, do Espírito Santo Pereira A, Mancini SD, Lipponen J, Vilaplana F (2023) Lignocellulosic biomass from agricultural waste to the circular economy: a review with focus on biofuels, biocomposites and bioplastics. J Clean Prod 402:136815. 10.1016/j.jclepro.2023.136815

[CR57] Nieves RA, Ehrman CI, Adney WS, Elander RT, Himmel ME (1998) Survey and analysis of commercial cellulase preparations suitable for biomass conversion to ethanol. World J Microbiol Biotechnol 14(2):301–304. 10.1023/A:1008871205580

[CR58] Okada G (1985) Purification and properties of a cellulase from *Aspergillus niger*. Agric Biol Chem 49(5):1257–1265. 10.1080/00021369.1985.10866894

[CR59] Government RM, Olowokere JA, Odineze CM, Anidobu CO, Yerima EA, Nnaemeka BI (2019) Influence of soaking time and sodium hydroxide concentration on the chemical composition of treated mango seed shell flour for composite application. J Appl Sci Envir Manag 23(1):21–28. 10.4314/jasem.v23i1.3

[CR60] Østby H, Hansen LD, Horn SJ, Eijsink VGH, Várnai A (2020) Enzymatic processing of lignocellulosic biomass: principles, recent advances and perspectives. J Ind Microbiol Biotechnol 47(9–10):623–657. 10.1007/s10295-020-02301-8 32840713 10.1007/s10295-020-02301-8 PMC7658087

[CR61] Park S, Baker JO, Himmel ME, Parilla PA, Johnson DK (2010) Cellulose crystallinity index: measurement techniques and their impact on interpreting cellulase performance. Biotechnol Biofuels 3(1):10. 10.1186/1754-6834-3-10 20497524 10.1186/1754-6834-3-10 PMC2890632

[CR62] Payne CM, Knott BC, Mayes HB, Hansson H, Himmel ME, Sandgren M, Ståhlberg J, Beckham GT (2015) Fungal cellulases. Chem Rev 115(3):1308–1448. 10.1021/cr500351c 25629559 10.1021/cr500351c

[CR63] Peciulyte A, Karlström K, Larsson PT, Olsson L (2015) Impact of the supramolecular structure of cellulose on the efficiency of enzymatic hydrolysis. Biotechnol Biofuels 8(1):56. 10.1186/s13068-015-0236-9 25870653 10.1186/s13068-015-0236-9 PMC4394567

[CR64] Pel HJ, de Win JH, Archer DB, Dyer PS, Hofmann G, Schaap PJ, Turner G, de Vries RP, Albang R, Albermann K, Andersen MR (2007) Genome sequencing and analysis of the versatile cell factory *Aspergillus niger* CBS 513.88. Nat Biotechnol 25(2):221–231. 10.1038/nbt1282 17259976 10.1038/nbt1282

[CR65] Puchart V, Biely P (2023) Microbial xylanolytic carbohydrate esterases. Essays Biochem 67(3):479–491. 10.1042/ebc20220129 36468678 10.1042/EBC20220129

[CR66] Ralph J, Quideau S, Grabber JH, Hatfield RD (1994) Identification and synthesis of new ferulic acid dehydrodimers present in grass cell walls. J Chem Soc, Perkin Trans 1 23:3485–3498. 10.1039/P19940003485

[CR67] Ravichandra K, Balaji R, Devarapalli K, Batchu UR, Thadikamala S, Banoth L, Pinnamaneni SR, Prakasham RS (2023) Enzymatic production of prebiotic xylooligosaccharides from sorghum (*Sorghum bicolor* (L.) xylan: value addition to sorghum bagasse. Biomass Conv Bioref 13(12):11131–11139. 10.1007/s13399-021-02216-z

[CR68] Sannigrahi P, Kim DH, Jung S, Ragauskas A (2011) Pseudo-lignin and pretreatment chemistry. Energy Environ Sci 4(4):1306–1310. 10.1039/C0EE00378F

[CR69] Shei JC, Fratzke AR, Frederick MM, Frederick JR, Reilly PJ (1985) Purification and characterization of endo-xylanases from *Aspergillus niger*. II. An enzyme of pl 4.5. Biotechnol Bioeng 27(4):533–538. 10.1002/bit.260270421 18553704 10.1002/bit.260270421

[CR70] Sherief AA (1990) Separation and some properties of an endo-1,4-beta-D-xylanase from *Aspergillus flavipes*. Acta Microbiol Hung 37(3):301–306 2100903

[CR71] Siacor FDC, Lobarbio CFY, Taboada EB (2021a) Pretreatment of mango (*Mangifera indica L.* Anacardiaceae) seed husk for bioethanol production by dilute acid treatment and enzymatic hydrolysis. Appl Biochem Biotechnol 193(5):1338–1350. 10.1007/s12010-020-03387-7 32888162 10.1007/s12010-020-03387-7

[CR72] Siacor FDC, Tabañag IDF, Lobarbio CFY, Taboada EB (2021b) Effects of aqueous ethanol concentration and solid-to-liquid ratio in the extraction of organosolv lignin from mango (*Mangifera indica* L.) seed husk. Sci Technol Asia 26(2):34–45

[CR73] Sternberg D, Vijayakumar P, Reese ET (1977) β-glucosidase: microbial production and effect on enzymatic hydrolysis of cellulose. Can J Microbiol 23(2):139–147. 10.1139/m77-020 837251 10.1139/m77-020

[CR74] Sulyman AO, Igunnu A, Malomo SO (2020) Isolation, purification and characterization of cellulase produced by *Aspergillus niger* cultured on *Arachis hypogaea* shells. Heliyon. 10.1016/j.heliyon.2020.e05668 33319112 10.1016/j.heliyon.2020.e05668 PMC7723808

[CR75] Van Dyk JS, Pletschke BI (2012) A review of lignocellulose bioconversion using enzymatic hydrolysis and synergistic cooperation between enzymes—factors affecting enzymes, conversion and synergy. Biotechnol Adv 30(6):1458–1480. 10.1016/j.biotechadv.2012.03.002 22445788 10.1016/j.biotechadv.2012.03.002

[CR76] Várnai A, Huikko L, Pere J, Siika-aho M, Viikari L (2011) Synergistic action of xylanase and mannanase improves the total hydrolysis of softwood. Bioresour Technol 102(19):9096–9104. 10.1016/j.biortech.2011.06.059 21757337 10.1016/j.biortech.2011.06.059

[CR77] Visser H, Joosten V, Punt PJ, Gusakov AV, Olson PT, Joosten R, Bartels J, Visser J, Sinitsyn AP, Emalfarb MA, Verdoes JC (2011) Development of a mature fungal technology and production platform for industrial enzymes based on a *Myceliophthora thermophila* isolate, previously known as *Chrysosporium lucknowense* C1. Ind Biotechnol 7(3):214–223. 10.1089/ind.2011.7.214

[CR78] Vlasenko E, Schulein M, Cherry J, Xu F (2010) Substrate specificity of family 5, 6, 7, 9, 12, and 45 endoglucanases. Bioresour Technol 101(7):2405–2411. 10.1016/j.biortech.2009.11.057 20006928 10.1016/j.biortech.2009.11.057

[CR79] Wang D, Zhang L, Zou H, Wang L (2018) Secretome profiling reveals temperature-dependent growth of *Aspergillus fumigatus*. Sci China Life Sci 61(5):578–592. 10.1007/s11427-017-9168-4 29067645 10.1007/s11427-017-9168-4

[CR80] Ximenes EA, Felix CR, Ulhoa CJ (1996) Production of cellulases by *Aspergillus fumigatus* and characterization of one β-glucosidase. Curr Microbiol 32(3):119–123. 10.1007/s002849900021

[CR81] Yadav SPS, Paudel P (2022) The process standardizing of mango (*Magnifera indica*) seed kernel for its value addition: a review. RFNA 3(1):6–12. 10.26480/rfna.01.2022.06.12

[CR82] Yang J, Kim JE, Kim JK, Lee SH, Yu J-H, Kim KH (2017) Evaluation of commercial cellulase preparations for the efficient hydrolysis of hydrothermally pretreated empty fruit bunches. BioResources 12(4):7834–7840. 10.15376/biores.12.4.7834-7840

[CR83] Yu S, Li Z, Wang Y, Chen W, Fu L, Tang W, Chen C, Liu Y, Zhang X, Ma L (2015) High-level expression and characterization of a thermophilic β-mannanase from *Aspergillus niger* in *Pichia pastoris*. Biotechnol Lett 37(9):1853–1859. 10.1007/s10529-015-1848-7 25967034 10.1007/s10529-015-1848-7

[CR84] Zhang S, Zhao S, Shang W, Yan Z, Wu X, Li Y, Chen G, Liu X, Wang L (2021) Synergistic mechanism of GH11 xylanases with different action modes from *Aspergillus niger* An76. Biotechnol Biofuels 14(1):118. 10.1186/s13068-021-01967-1 33971954 10.1186/s13068-021-01967-1 PMC8112042

