# Supplementary material for: Utilisation of mango seed husk for the production of phenolic compounds and glucose with C1184 enzyme preparation reveals the role of glucuronoyl esters in lignin–carbohydrate linkages in biomass recalcitrance
Source: Bioresour Bioprocess. 2025 Dec 27;12(1):155. doi: 10.1186/s40643-025-00989-z (PMC12743729; doi:10.1186/s40643-025-00989-z)
Supplement: Supplementary file 1 — Supplementary Material 1 [file 40643_2025_989_MOESM1_ESM.docx]

**Supplementary data for**

**Utilisation of mango seed husk for the production of phenolic compounds and glucose with C1184 enzyme preparation reveals the role of glucuronoyl esters in lignin–carbohydrate linkages in biomass recalcitrance**

Mpho Stephen Mafa^a,^*, Mamosela Marriam Mohotloane^a^, Orbett Alexander^b^, Mathapelo Hope Masilo^a^, Anikó Várnai^c,^*

^a^Carbohydrates and Enzymology Laboratory (CHEM-LAB), Department of Plant Sciences, University of the Free State, P.O. Box 339, Bloemfontein 9300, South Africa

^b^Department of Chemistry, University of the Western Cape, Bellville, Cape Town 7535, South Africa

^c^Norwegian University of Life Sciences (NMBU), Faculty of Chemistry, Biotechnology and Food Science, P.O. Box 5003, N-1432 Aas, Norway

*Corresponding authors: [mafams@ufs.ac.za](mailto:mafams@ufs.ac.za); [aniko.varnai@nmbu.no](mailto:aniko.varnai@nmbu.no)


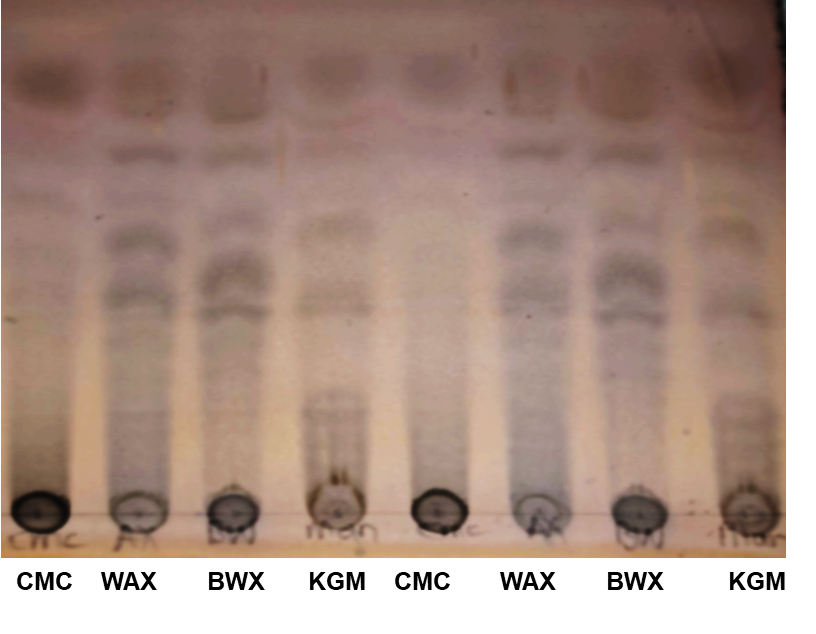


**Figure S1.** Thin-layer chromatography analysis showing oligosaccharides produced from carboxymethylcellulose (CMC), wheat arabinoxylan (WAX), beechwood xylan (BWX) and konjac glucomannan (KGM) by the C1184 preparation, demonstrating that it contains cellulolytic, xylanolytic and mannanolytic enzymes. The left and right sides of the figure show samples from technical duplicates for each substrate.


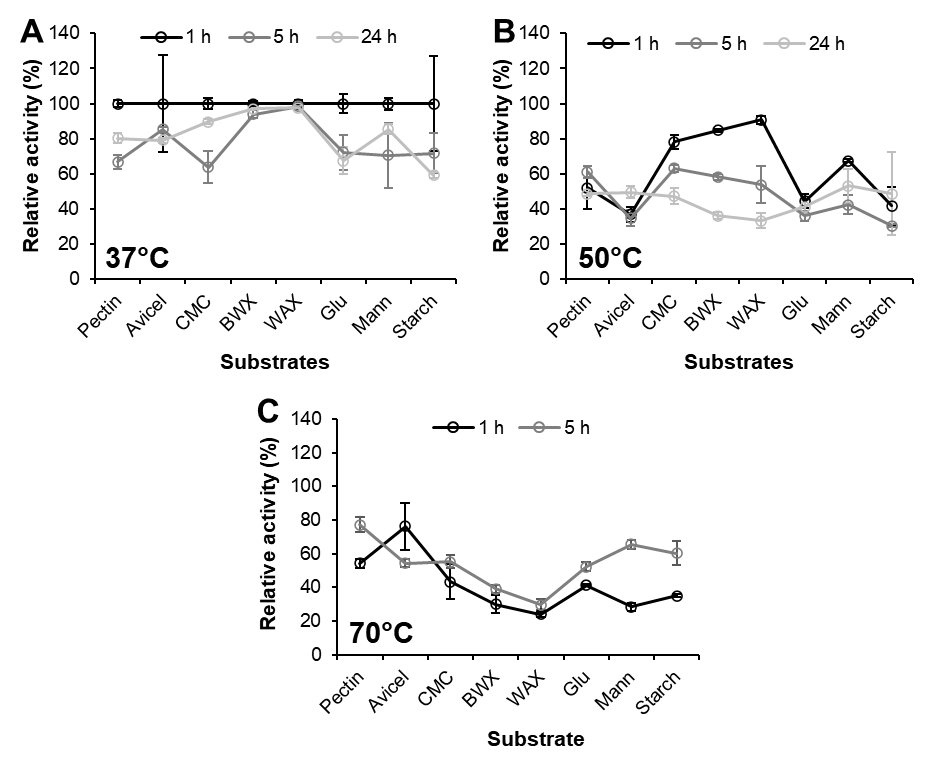


**Figure S2.** Temperature optima of the C1184 preparation assayed using different polysaccharide substrates, i.e., pectin from apple, Avicel, carboxymethylcellulose (CMC), beechwood xylan (BWX), wheat arabinoxylan (WAX), algal β-(1→3)-glucan (Glu), konjac glucomannan (Mann) and soluble starch. Three temperature regimes were used: 37°C **(A)**, 50°C **(B)** and 70°C **(C)**. The C1184 preparation was incubated at these temperatures for 1 (black lines), 5 (dark grey lines) and 24 h (light grey lines). Activity values are relative to the activity values measured at 37°C after 1 h. The experiments were performed in triplicates; the values represent means and the error bars standard deviation.


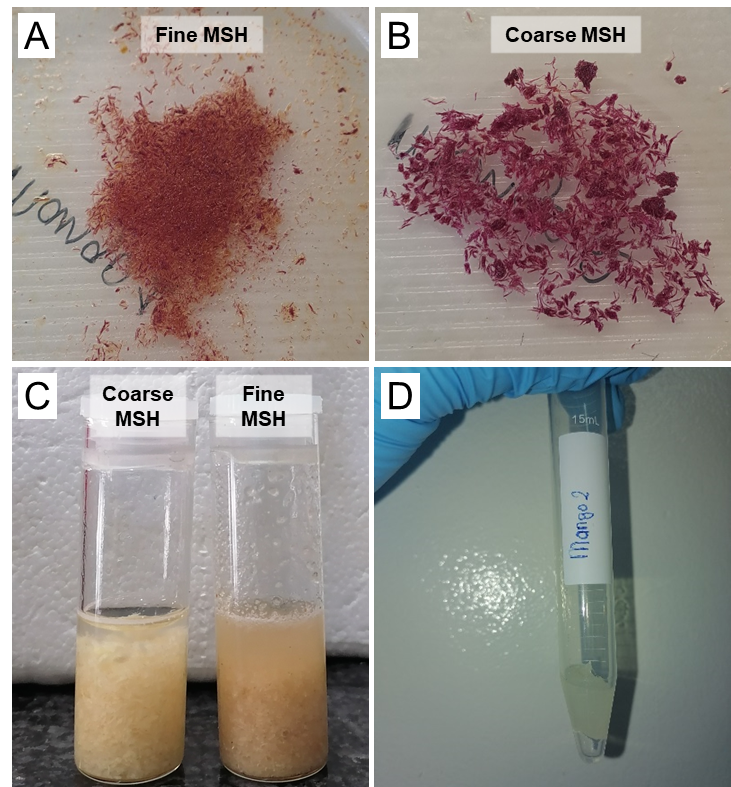


**Figure S3. Visual characterisation of mango seed husk (MSH) fractions.** Panels **A** and **B** show the fine **(A)** and course **(B)** fractions of MSH after phloroglucinol staining. The light pinkish-red colour in panel **A** indicates lower lignin content in the fine fraction, while the deep pinkish-purple colour in panel **B** indicates higher lignin content in the course fraction. Panel **C** shows the dried fine (to the right) and course (to the left) fractions of MSH after rehydration. Note the more hydrophobic nature of the coarse fraction (i.e., the sample sedimented) and the more hydrophilic nature of the fine fraction (i.e., the sample did not sediment even 48 h after the rehydration process). Panel **D** shows that a gel-like structure (often referred to as biofilm) that the solubles dissolved during after alkali pretreatment of the coarse fraction formed after ethanol precipitation. Similar gel formation was not observed for the fine fraction.


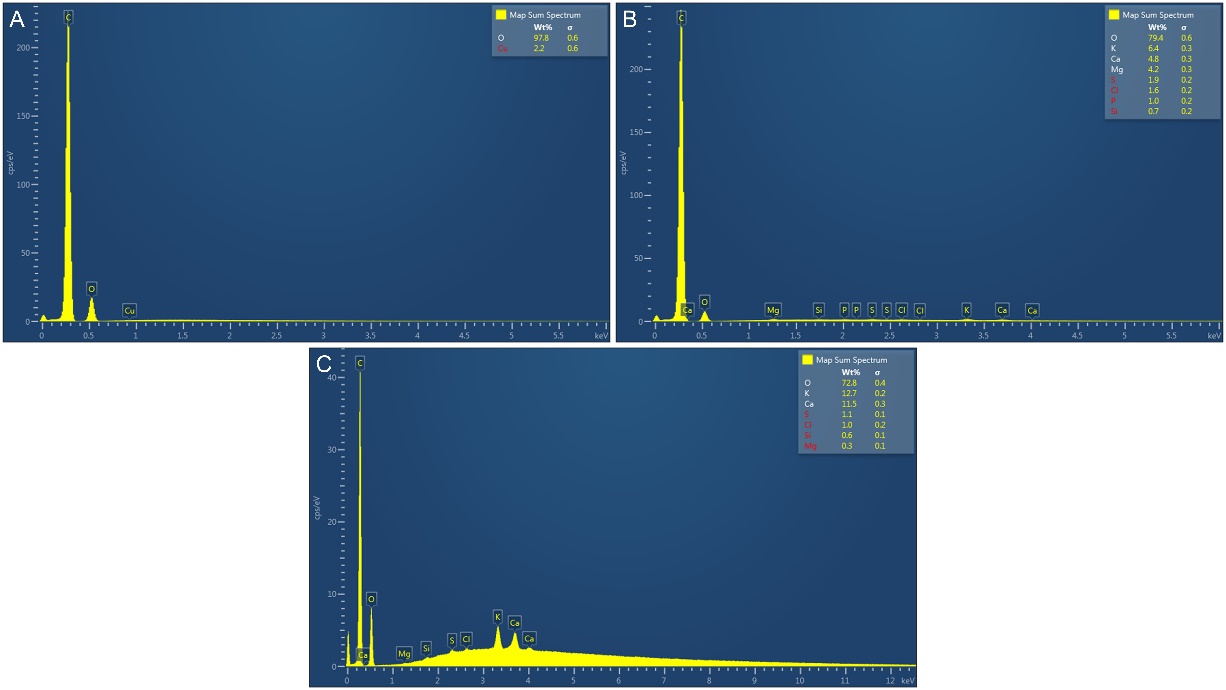


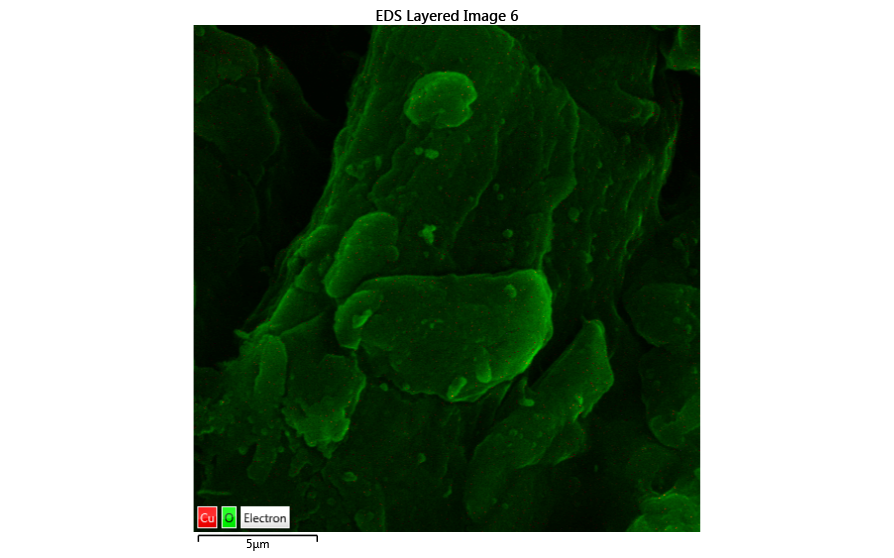

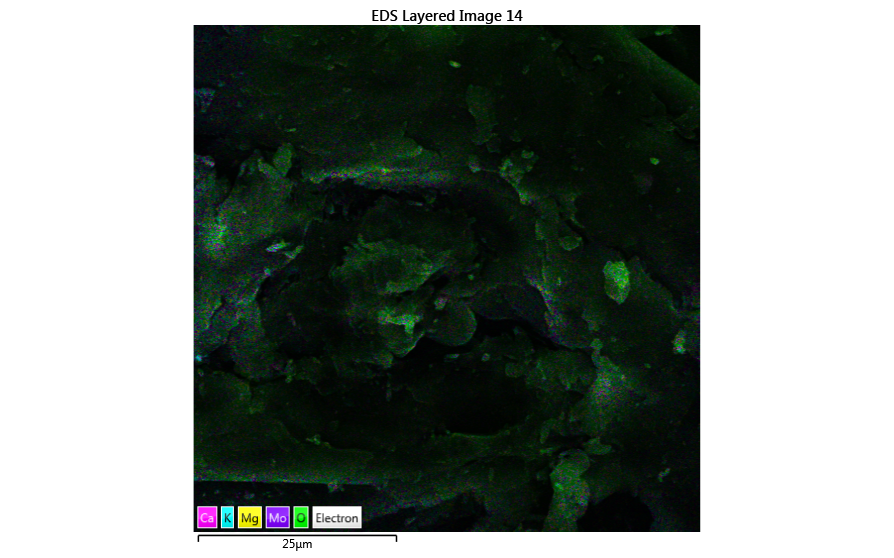

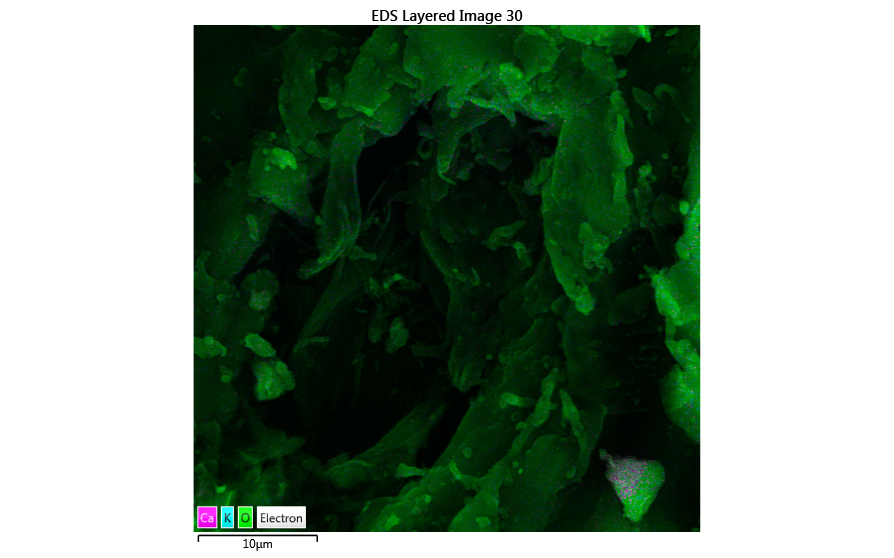


F

E

D

**Figure S4:** Elemental analysis of Avicel **(A)**, fine mango seed husk **(B)** and coarse mango seed husk **(C)**. The element's weight % indicated by white text is abundant, and red text indicates trace amounts. The example of the SEM elemental maps shows that the elements lead to the granulated particle on the biomass surface; **D**, **E** and **F** represent the Avicel, fine and rough mango seed husk.
